# RNA-Sequencing Reveals Differentially Expressed Rice Genes Functionally Associated with Defense against BPH and WBPH in RILs Derived from a Cross between RP2068 and TN1

**DOI:** 10.1186/s12284-021-00470-3

**Published:** 2021-03-06

**Authors:** Dhanasekar Divya, Nihar Sahu, P. Sairam Reddy, Suresh Nair, J. S. Bentur

**Affiliations:** 1 Agri Biotech Foundation, Rajendranagar, Hyderabad, 500030 India; 2 Present Address: Urbankisaan Farms Pvt Ltd, 4th Floor, 36 urban center, Rd. 36, CBI colony, Jubilee Hills, Hyderabad, 500033 India; 3 International Centre for Genetic Engineering and Biotechnology, Aruna Asaf Ali Marg, New Delhi, 110067 India

**Keywords:** Rice planthoppers, Sympatric pests, miRNA, *Nilaparvata lugens*, *Sogatella furcifera*, RNA seq analysis, Differential expression of genes, Plant resistance, Insect-plant interactions

## Abstract

**Background:**

Rice is staple food for over two billion people. Planthoppers like BPH and WBPH occur together in most of rice growing regions across Asia and cause extensive yield loss by feeding and transmission of disease-causing viruses. Chemical control of the pest is expensive and ecologically disastrous; breeding resistant varieties is an acceptable option. But most of such efforts are focused on BPH with an assumption that these varieties will also be effective against WBPH. No critical studies are available to understand rice resistance, common or otherwise, against these two planthoppers.

**Results:**

Our studies aimed to understand the defense mechanisms in rice line RP2068 against BPH and WBPH through RNA sequencing analysis of a RIL line TR3RR derived from the cross TN1 (susceptible) and RP2068 (resistant) after infestation with BPH or WBPH. Results revealed higher number of differentially expressed genes (DEGs) in BPH infested plants than in WBPH infested plants when compared with the uninfested plants. These DEGs could be grouped into UPUP, DNDN, UPDN and DNUP groups based on whether the DEGs were up (UP) or down (DN) regulated against BPH and WBPH, respectively. Gene ontology analysis, specially of members of the last two groups, revealed differences in plant response to the two planthoppers. Abundance of miRNAs and detection of their target genes also indicated that separate sets of genes were suppressed or induced against BPH and WBPH. These results were validated through the analysis of expression of 27 genes through semi-quantitative and quantitative real-time RT-PCR using a set of five RILs that were genetically identical but with different reaction against the two planthoppers. Coupled with data obtained through pathway analysis involving these 27 genes, expression studies revealed common and differential response of rice RP2068 against BPH and WBPH. Trehalose biosynthesis, proline transport, methylation were key pathways commonly upregulated; glucosinolate biosynthesis, response to oxidative stress, proteolysis, cytokinesis pathways were commonly down regulated; photosynthesis, regulation of transcription, expression and transport of peptides and defense related pathways were exclusively upregulated against WBPH; MYB transcription factor mediated defense induction was exclusive to BPH.

**Conclusion:**

Rice defense against the two sympatric planthoppers: BPH and WBPH has distinct features in RP2068. Hence, a conscious combination of resistance to these two pests is essential for effective field management.

**Supplementary Information:**

The online version contains supplementary material available at 10.1186/s12284-021-00470-3.

## Background

Rice (*Oryza sativa* L.) is one of the most important staple food crops of the world. Significant amount of the rice production is lost annually due to biotic stresses, of which about 25% is attributed to the insect pests (Savary et al. 2000). Planthoppers, such as brown planthopper (BPH) [*Nilaparvata lugens* (Stål)] and whitebacked planthopper (WBPH) [*Sogatella furcifera* (Horváth)] have again attained peak pest status in Asia since the beginning of this century (Bentur and Viraktamath 2008; Bottrell and Schoenly 2012). These insect pests inflict losses not only by direct sap sucking from rice but also by acting as vectors of disease-causing plant viruses (Zhou et al. 2008). Currently, these pests are being managed by the farmers largely through heavy use of environmentally harmful synthetic insecticides (Heong and Hardy 2009). However, breeding rice varieties resistant to these pests would be an environmentally safe and ecologically acceptable alternative approach to manage these pests (Brar et al. 2009).

Studies were initiated during 1960s to identify, characterize and utilize rice land races with resistance to BPH and WBPH (Pathak et al. 1969; IRRI 1979; Heinrichs et al. 1985). So far about 40 major genes and 72 QTLs conferring resistance to BPH and 19 major genes and 75 QTLs conferring resistance to WBPH have been reported from cultivated rice and its wild relatives (Fujita et al. 2013, Ling and Weilin 2016; Du et al. 2020; Haliru et al. 2020). Using some of these genes or combinations thereof, several rice varieties possessing BPH resistance have been developed and released for cultivation since 1980s (Khush and Brar 1991; Brar et al. 2009). With advent of molecular marker technology almost all the BPH R genes have been mapped with linked markers (Du et al. 2020, Haliru et al. 2020). It has been observed that 30 R genes are located in six clusters on four chromosomes i.e. 3, 4, 6 and 12. The cluster on chromosome 12 is reported to be the most dense with 8 genes located within a 5 MB region (Du et al. 2020). So far, 14 of these R genes have been cloned through map-based approach (Zhao et al. 2016; Guo et al. 2018). Through sequencing of R genes from different reported sources, Zhao et al. (2016) reported *Bph9, Bph1, bph7*, *Bph10* and *Bph21* to be different alleles of the same gene. Characterization of these cloned R genes suggested 10 of the genes to belong to NBS-LRR family of resistance genes while two (*Bph3* and *Bph15*) represented lectin receptor kinases; *bph29* has a B3 DNA binding domain and *Bph32* is novel with an unknown SCR domain (Du et al. 2020). Thus, a fair degree of diversity, in terms of functional domains, is represented by the different BPH R genes and consequently in their mode of action. This information coupled with availability of linked molecular markers for many BPH R genes (Hu et al. 2016) has led to a spurt in molecular breeding aimed at pyramiding BPH R genes to provide durable resistance (Liu et al. 2015; Wang et al. 2015; Fan et al. 2017; Wang et al. 2018; Han et al. 2018; Jiang et al. 2018). In contrast, less intense work is reported on WBPH resistance genes. Of the 12 major WBPH resistance genes reported, two being introgressed from the wild rice - *Oryza officinalis,* nine gene have been mapped with linked markers (Ramesh et al. 2014; Du et al. 2020). However, none of the genes has been cloned and characterized.

BPH and WBPH are sympatric species occurring together in almost all rice ecologies (Horgan et al. 2020). Both are phloem feeders and transmit rice viruses. While BPH is reported to transmit rice ragged stunt virus and grassy stunt virus, WBPH has been reported to transmit southern rice black streaked dwarf virus (Pu et al. 2012). Both are capable of long-distance migration (Otuka et al. 2008). Phenologically, WBPH colonizes the crop early during vegetative stage while BPH appears at late active tillering stage. Interspecific competition generally results in dominance of BPH as the crop grows. While BPH is monophagous confined to rice (*Oryza*), having shifted its host from *Leersia* about 0.25 million years ago (Jones et al. 1996; Sezer and Butlin 1998), WBPH is oligophagous capable of feeding and surviving on plants from several genera of the family Poaceae (Zhou et al. 2013). Genomes of both insects have been sequenced (Xue et al. 2014; Wang et al. 2017) and of the two, BPH is reported to possess a larger genome (1141 MB) and the size of the WBPH genome has been estimated to be 720 MB. Despite apparent similarities between these two planthoppers, host-plant resistance is not common across these. It has been reported that BPH R genes reported thus far are not effective against WBPH, though *Bph3* (Liu et al. 2015) and *Bph6* (Guo et al. 2018) have been claimed to be effective against WBPH also. Further, several land races and breeding lines have been reported to be resistant to both BPH and WBPH (Heinrichs et al. 1985; Bentur et al. 2011). Genetic analyses of some of these land races and breeding lines e.g. Sinna Sivappu (Ramesh et al. 2014) and ADR52 (Srinivasan et al. 2015) indicate that loci conferring resistance to BPH in these donor parents are different from those responsible for WBPH resistance. From the point of view of pest management, cultivating rice varieties resistant to BPH alone may prove disastrous as WBPH, in the absence of BPH, could fill in the vacuum and pose a more severe threat than what it would in the concomitant presence of BPH (Bottrell and Schoenly 2012). Therefore, from a scientific standpoint, it is important to ascertain how defense pathways that operate against BPH are different from those operating against WBPH.

Omics tools have been successfully employed to investigate molecular pathways triggered or suppressed in rice genotypes when challenged by BPH. Zhang et al. (2004) studied expression profiles of a limited number of defense genes in a susceptible (MH63) and a resistant (B5) rice lines following BPH infestation and observed that genes related to signaling pathways, oxidative stress/apoptosis, wound-response, drought-inducible and pathogen-related proteins were up-regulated in the resistant genotype. Similarly, employing suppressive subtraction hybridization (SSH) tools, Yuan et al. (2005) identified 25 differentially accumulated cDNAs of genes representing several functional categories such as cell wall proteins, protein folding and degradation, protein–protein interactions and/or signal transduction, lipid metabolism, stress response, and transport facilitation in a BPH susceptible cultivar Minghui63 at 32 h after infestation. Again, using an SSH approach, Wang et al. (2005), also identified 21 differentially expressed genes related to wound and stress tolerance in B5. Wang et al. (2012) employed microarray analysis to demonstrate differential expression of transcription factor (TF) genes in the resistant rice landrace Rathu Heenati and the susceptible TN1 at 24 h upon infestation with BPH. They identified 13 TF genes induced in the resistant cultivar. Li et al. (2017) also studied BPH resistance in Rathu Heenati through microarray analysis and concluded that salicylic acid plays a key role in the resistance process. Recent studies have focused on the role of miRNAs in conferring BPH resistance in rice (Wu et al. 2017; Ge et al. 2018; Dai et al. 2019). Using an approach combining both microRNA and transcriptome analyses Tan et al. (2020) identified 29 key miRNAs and 20 candidate genes regulating *Bph6*-mediated resistance in a transgenic rice line. Wei et al. (2009) adopted a proteomics approach to understand BPH resistance in a rice line with *Bph15* gene and concluded that it has a different defense mechanism which involves Gns5 and the glycine cleavage system H-protein. Kang et al. (2019) studied the metabolite profiles in three rice varieties TN1, IR36 and IR56 following feeding by BPH to get an understanding of the metabolic mechanism of rice resistance. The only report, thus far, on global expression profiling of resistance pathway genes in rice against WBPH identified four key genes, located on chromosome 6, to be involved in ovicidal response of the rice variety CJ06 to WBPH infestation (Yang et al. 2014). Li et al. (2020) made a comparative transcriptome analysis of defense response of rice to BPH and striped stem borer infestation. However, to the best of our knowledge, no study has been reported that aims to understand differential resistance mechanism in genetically related rice genotypes against BPH and WBPH through expression profiling. In this study, we used F_14_ RILs derived from a cross between the rice breeding line RP2068-18-3-5 (RP2068; resistant to both BPH and WBPH), and TN1 (susceptible to both the planthoppers). Using an RNA-seq approach, specific transcripts displaying differential induction were identified in the RILs and subsequently, gene expression of 27 selected target genes was validated to understand and obtain useful insights into the molecular process of rice resistance against two of its major pests i.e. BPH and WBPH. The results of the current study indicated that rice defense against WBPH is of lower order with emphasis on tolerance as against BPH where the emphasis is on antibiosis.

## Results

### Performance of RILs against BPH and WBPH

**Fig. 1:**
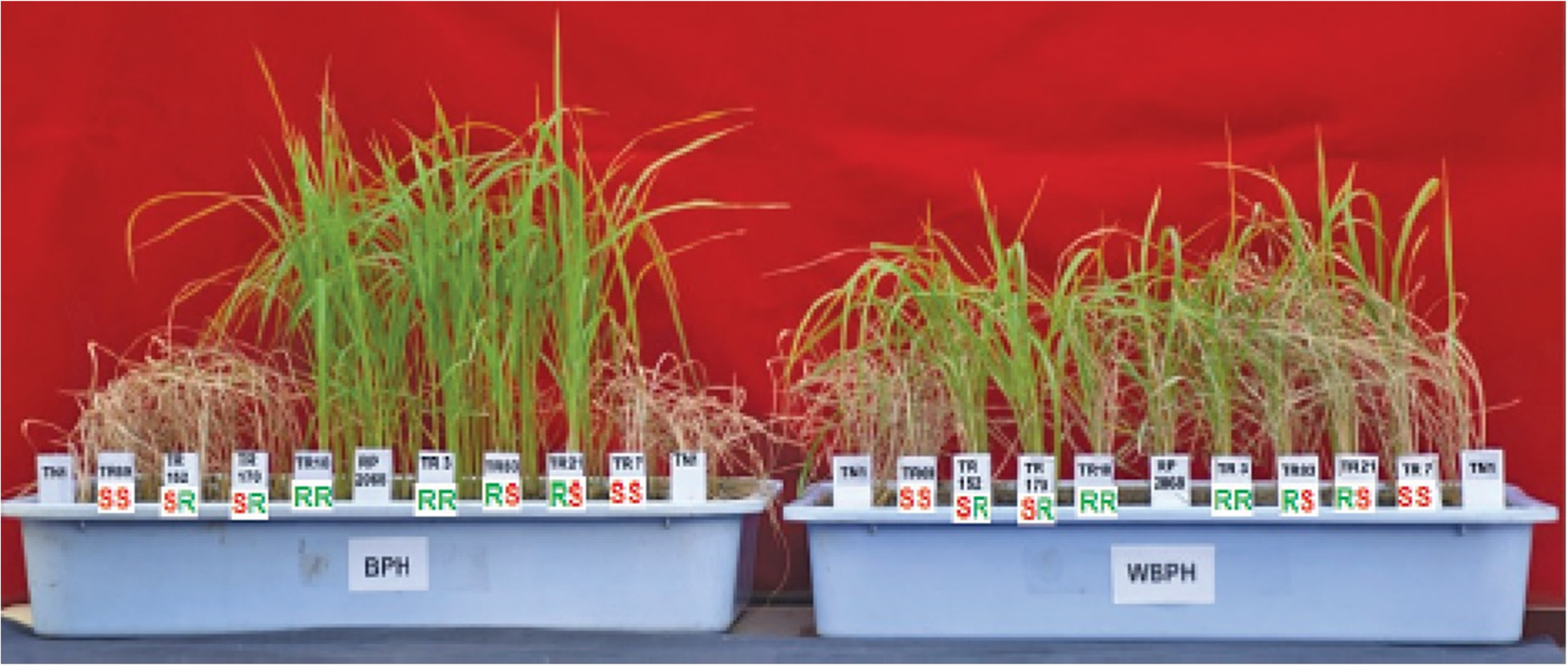
Performance of RILs derived from a cross between TN1 and RP2068-18-3-5 against BPH and WBPH in standard seedbox screening test in the greenhouse. RILs in the trays from L to R are TN1(S parent), TR24SS, TR152SR, TR173SR, TR94RR, RP2068 (R parent), TR3RR, TR145RS, TR21RS, TR7SS, TN1 (S parent). Left tray was exposed to BPH while right tray was exposed to WBPH

**Fig. 2:**
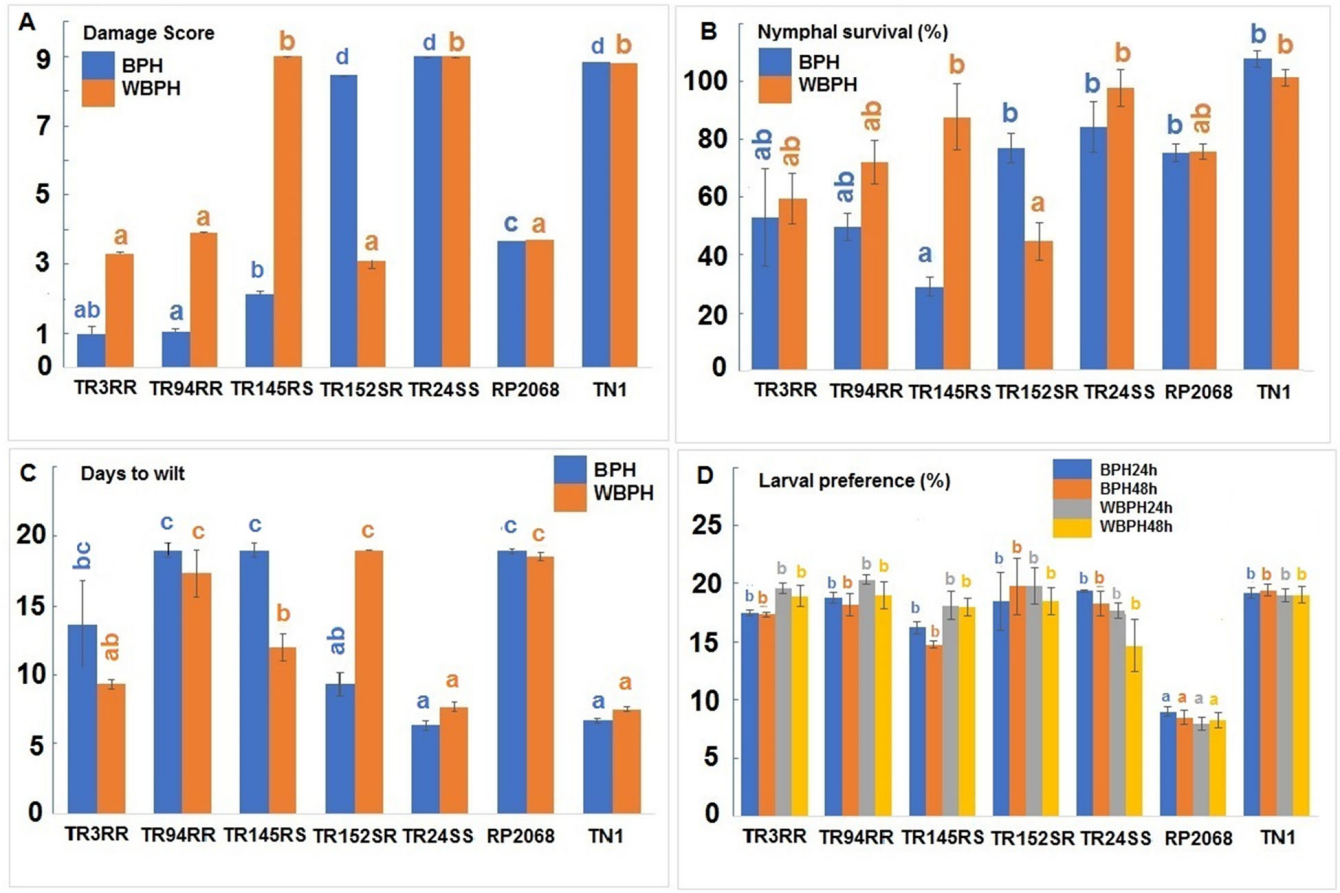
Performance of five selected RILs and the parents in four phenoptyping tests against BPH and WBPH. Bars indicate mean ± SEM; means with same letter (in same color) are not significantly different (paired ‘t’ test, *P* < 0.05). Rice lines are mentioned on the X-axis. Damage scores are indicated on the Y-axis

All the 180 RILs were subjected to four phenotypic tests against BPH and WBPH, separately. In each of the tests, RILs could be grouped into RR with resistance to both the planthoppers; RS with resistance to BPH only; SR with resistance to WBPH only and SS with no resistance against both the planthoppers (Table 1). Thus, resistance in RP2068 against BPH was independent of resistance against WBPH. Under standard seedbox screening test (SSST) nine RILs displayed RR reaction; 16 were RS; 28 were SR and the remaining were SS. While resistance to BPH among RILs segregated in 25R:155S suggesting possible involvement of three genes (χ^2^ = 0.343; *P* = 0.558), resistance to WBPH among RILs segregated in 37R:143S suggesting two gene involvement (χ^2^ = 1.896; *P* = 0.168). Other phenotypic tests such as nymphal survival (antibiosis component of resistance), days to wilt (tolerance component) and nymphal preference (antixenosis component) also revealed similar trend of segregation of resistance among RILs against BPH and WBPH. Based on the overall performance (Fig. 1) and in all the four phenotypic tests (Fig. 2), five RILs: TR3RR, TR94RR, TR145RS, TR152SR and TR24SS were selected for the further studies. These selected RILs had a genetic similarity ranging from 44 to 84% based on screening results involving 137 molecular markers polymorphic between the parents (Supplementary Table S1). Further, based on marker polymorphism in respect of the two linked markers RM488 and RM11522 (Naik et al. 2018), four of the five RILs: TR3RR, TR94RR, TR145RS and TR152SR are likely to carry *Bph33* gene.

**Table 1:** Segregation of different traits of resistance against BPH and WBPH among RILs derived from the cross between TN1 X RP2068 observed in different phenotyping tests under greenhouse conditions

Test	Number of RILs under group			
	RR^a^	RS	SR	SS
Damage score (DS) in standard seedbox screening test (SSST)	9	16	28	127
Nymphal survival (%, NS)	8	22	33	117
Days to wilt (DW)	13	14	49	104
Nymphal preference at 24 h (%, NP24)	8	38	29	105
Nymphal preference at 48 h (%, NP48)	13	30	38	99

Threshold values for R in DS: ≤ 3.0 (BPH), ≤ 4.0 (WBPH); NS ≤ 60.0%; DW ≥ 10.0; NP ≤ 10%

^a^RR- resistant to both BPH & WBPH; RS- resistant to BPH only; SR- resistant to WBPH only; SS - susceptible to both BPH and WBPH

### RNA-Sequencing

RNA-seq data generated from the RIL TR3RR, an individual line representing F_14_ generation of a mapping population derived from a cross between TN1 and RP2068, produced 272 million raw reads from nine samples (Supplementary Table S2). Between 22 and 41 million raw reads were generated from individual samples. High quality clean reads of leaf sheath tissue samples were mapped to the reference genome of *O. sativa indica (Oryza sativa indica*; ASM465 build genome downloaded from the Ensembl Plants database) and the gene expression level was estimated. The variability among the biological replicates indicated R^2^ value to be 0.759 for BPH, 0.785 for WBPH and when both BPH and WBPH data were pooled R^2^ was 0.345 (Supplementary Fig. S1 A, B, C), indicating slight variability within the biological replicates for BPH and WBPH and moderate variability among the pooled data.

**Fig. 3:**
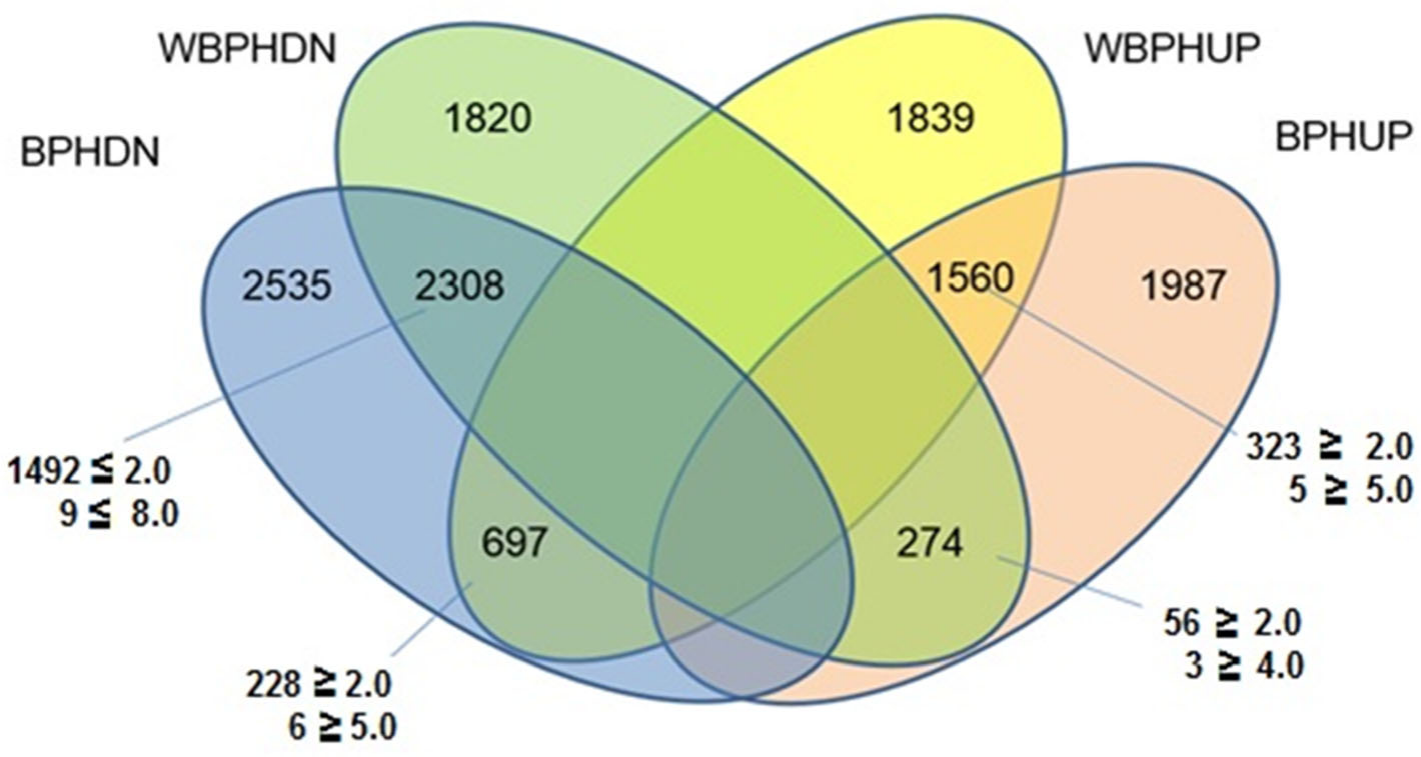
Venn diagram depicting differentially expressed transcripts in TR3RR RIL when challenged with either BPH or WBPH. Transcripts upregulated (UP) and downregulated (DN) upon BPH (BPH) or WBPH (WBPH) challenge are indicated. Number of transcripts in each group showing < 2- or > 2-fold increase in expression are indicated

A higher number of differentially expressed transcripts/genes (DEGs) was observed in the BPH infested plant tissue in comparison with the uninfested plant tissue than in the WBPH infested plant tissue in comparison with the uninfested plant tissue (BPH = 9361 vs WBPH = 8498). Among these, 3821 transcripts showed upregulation while 5540 showed downregulation against BPH in comparison with uninfested control tissue samples (Supplementary Data File No. 1). In the case of WBPH, 4096 transcripts showed upregulation and 4402 transcripts showed down regulation in comparison with uninfested control tissue samples (Supplementary Data File No. 2). These data when taken together (Fig. 3), a total of 1560 DEGs were found to be common among those upregulated (UPUP) against both the planthoppers and 2308 DEGs were commonly downregulated (DNDN). In all, 274 DEGs were upregulated against BPH while being downregulated against WBPH (UPDN). Conversely, 697 genes were downregulated against BPH but upregulated against WBPH (DNUP). Further, among the 1560 UPUP transcripts, 323 had more than two-fold induction while 5 DEGs had more than five-fold increase in transcript abundance against both the planthoppers. Likewise, 1492 of the DEGs among DNDN set displayed ≥ two-fold decrease in expression levels, while nine DEGs had ≥ eight-fold decrease. From the UPDN set, 56 transcripts showed ≥2-fold change in expression values. Of these, three transcripts showed 4-fold induction values. In the DNUP set, 228 transcripts had 2-fold change in values while six of these registered ≥5-fold change values. Some of the transcripts showing significant change in expression levels were traced back to known genes and their reported function (Table 2) which, subsequently, formed the basis for selection of genes for validation study using qRT-PCR. Significantly, several of the transcripts represented the smaller subunit of rRNA of endophytic bacterial flora. More studies are planned to note role of endophytic bacterial flora in planthopper-rice interactions.

**Table 2:** List of differentially expressed key genes selected^a^ for validation using qRT-PCR

S. No	Transcript ID	Locus ID	Gene name/function	Change in expression (X fold)	
				BPH	WBPH
	**UPUP**				
1	BGIOSGA011927	LOC_Os03g06850	B3 DNA binding domain	3.26	3.97
2	BGIOSGA005805	LOC_Os02g46640	HSP DnaJ protein	2.39	2.83
3	BGIOSGA014560	LOC_Os04g47190	Aminotransferase domain	2.26	2.87
4	BGIOSGA012548	LOC_Os03g21110	emp24/gp25L/p24	2.02	2.88
5	BGIOSGA011316	LOC_Os03g08800	CutA, chloroplast precursor	2.58	3.50
6	BGIOSGA023418	LOC_Os06g45090	Expressed protein	2.91	4.17
7	BGIOSGA018591	LOC_Os05g12170	Plant-specific domain TIGR01589	2.41	2.20
8	BGIOSGA030199	LOC_Os09g04160	Expressed protein	2.26	2.70
	**DNDN**				
9	BGIOSGA025818	LOC_Os07g33440.1	Cytochrome P450, putative,	−6.77	−6.31
10	BGIOSGA002685	LOC_Os01g03530	Multicopper oxidase domain	−5.59	−3.32
11	BGIOSGA025088	LOC_Os07g03710.1	SCP-like extracellular protein	−5.46	−5.40
12	BGIOSGA000957	LOC_Os01g50420.1 F	STE_MEKK_ste11_MAP3K.7 - STE kinases	−4.82	−3.13
13	BGIOSGA005235	LOC_Os01g73250	Abscisic acid stress and ripening	−4.05	−6.88
14	BGIOSGA001631	LOC_Os01g27210	Glutathione s transferase	−3.96	−2.76
15	BGIOSGA005232	LOC_Os01g73200	Peroxidase precursor	−3.61	−4.53
16	BGIOSGA029543	LOC_Os09g31430.1	Os9bglu30 - beta-glucosidase	−2.76	−8.60
	**UPDN**				
17	BGIOSGA013307	LOC_Os03g47140	Growth regulatory factor	2.24	−3.67
18	BGIOSGA013363	LOC_Os03g48490	Centromere protein	2.47	−4.67
19	BGIOSGA036408	LOC_Os12g13570.1	MYB family transcription factor	2.64	−2.92
	**DNUP**				
20	BGIOSGA004899	LOC_Os01g65210	Proton-dependent oligopeptide transport, putative	−3.38	2.38
21	BGIOSGA021302	LOC_Os06g23274	Zinc finger, C3HC4, domain	−3.89	2.39
22	BGIOSGA040594	LOC_Os10g01044	Isoflavone reductase, putative	−2.66	2.79
23	BGIOSGA014339	LOC_Os04g52700	Expressed protein	−3.51	3.34
24	BGIOSGA040670	LOC_Os05g18470	CRAL/TRIO domain	−2.41	2.36
25	BGIOSGA002552	LOC_Os01g01650.1	Isoflavone reductase like, putative	−2.10	3.16
26	BGIOSGA016833	LOC_Os04g45370.1	OsSAUR19 Auxin-responsive SAUR	−3.93	4.35
27	BGIOSGA034582	LOC_Os11g03440.1	MYB-like DNA-binding domain	−2.06	5.27

^a^Selection based on RNAseq data obtained for TR3RR when challenged with either BPH or WBPH

### Gene Ontology-Based Functional Annotation

**Fig. 4:**
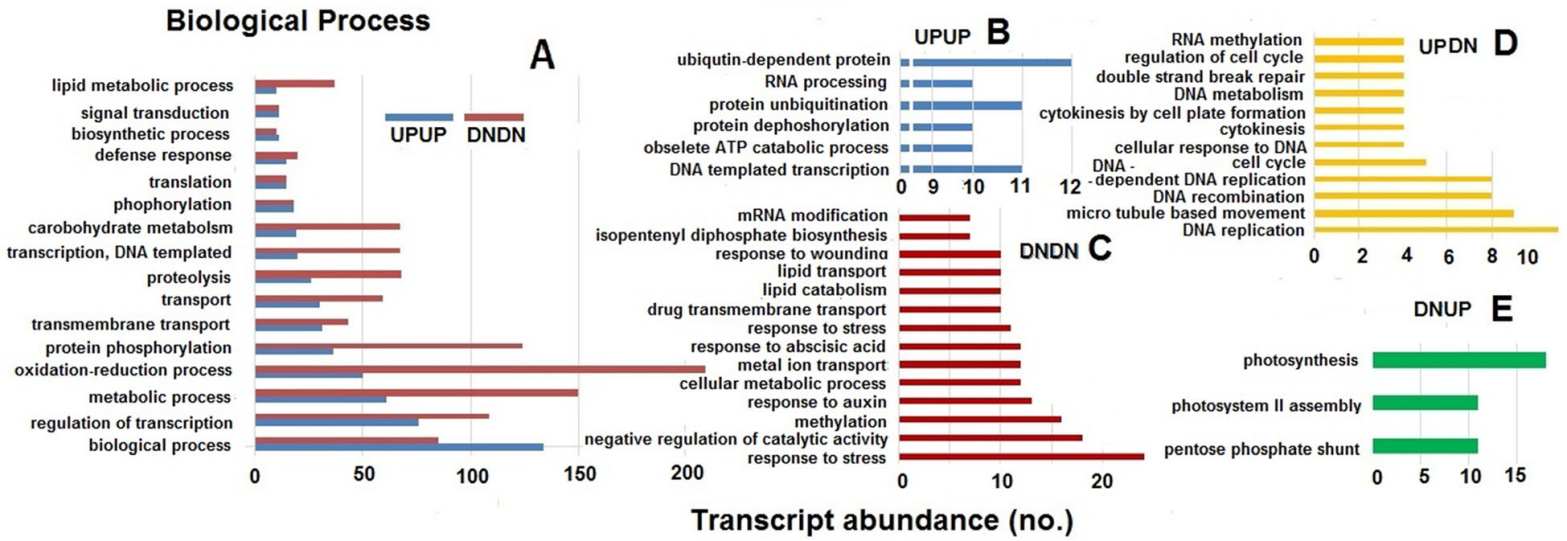
Abundance of transcripts of pathways under biological processes in differential expression groups in TR3RR plants infested with either BPH or WBPH. UPUP – upregulated against both BPH and WBPH; DNDN – down regulated against both BPH and WBPH; UPDN- upregulated against BPH but down regulated against WBPH; DNUP – down regulated against BPH but upregulated against WBPH

**Fig. 5:**
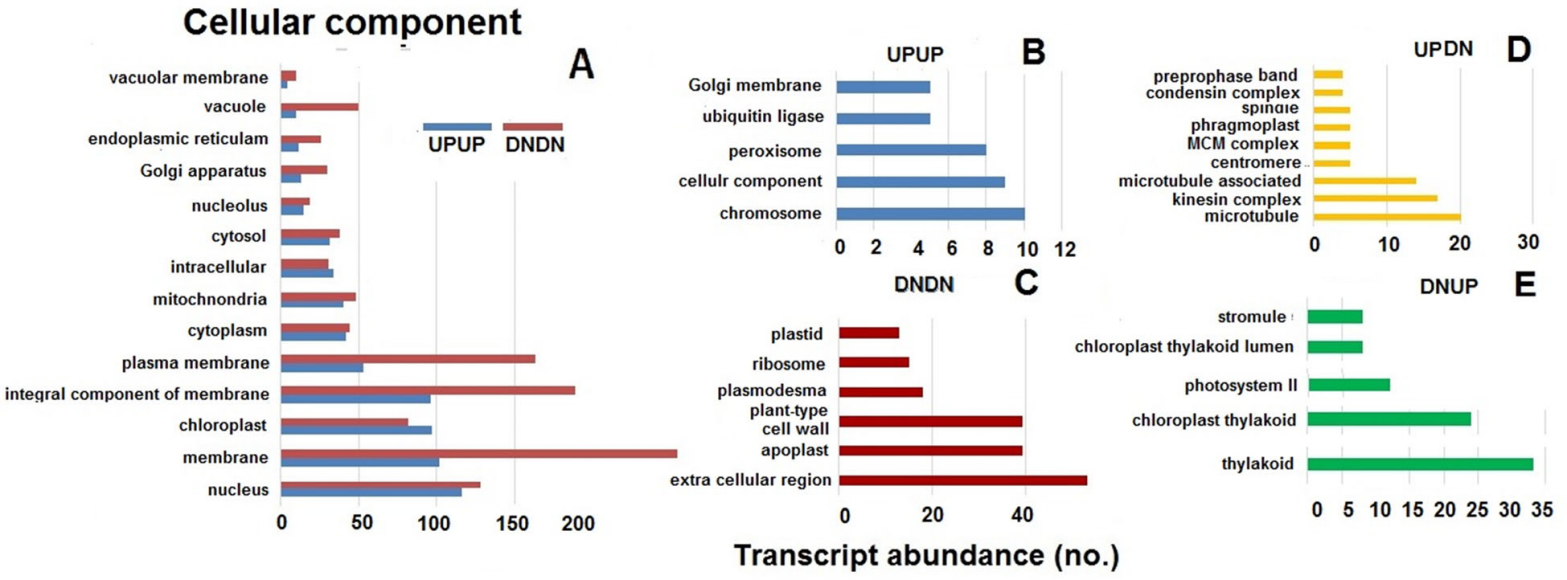
Abundance of transcripts of pathways under cellular component in differential expression groups in TR3RR plants infested with either BPH or WBPH. UPUP – upregulated against both BPH and WBPH; DNDN – down regulated against both BPH and WBPH; DNUP – down regulated against BPH but upregulated against WBPH; UPDN- upregulated against BPH but down regulated against WBPH

**Fig. 6:**
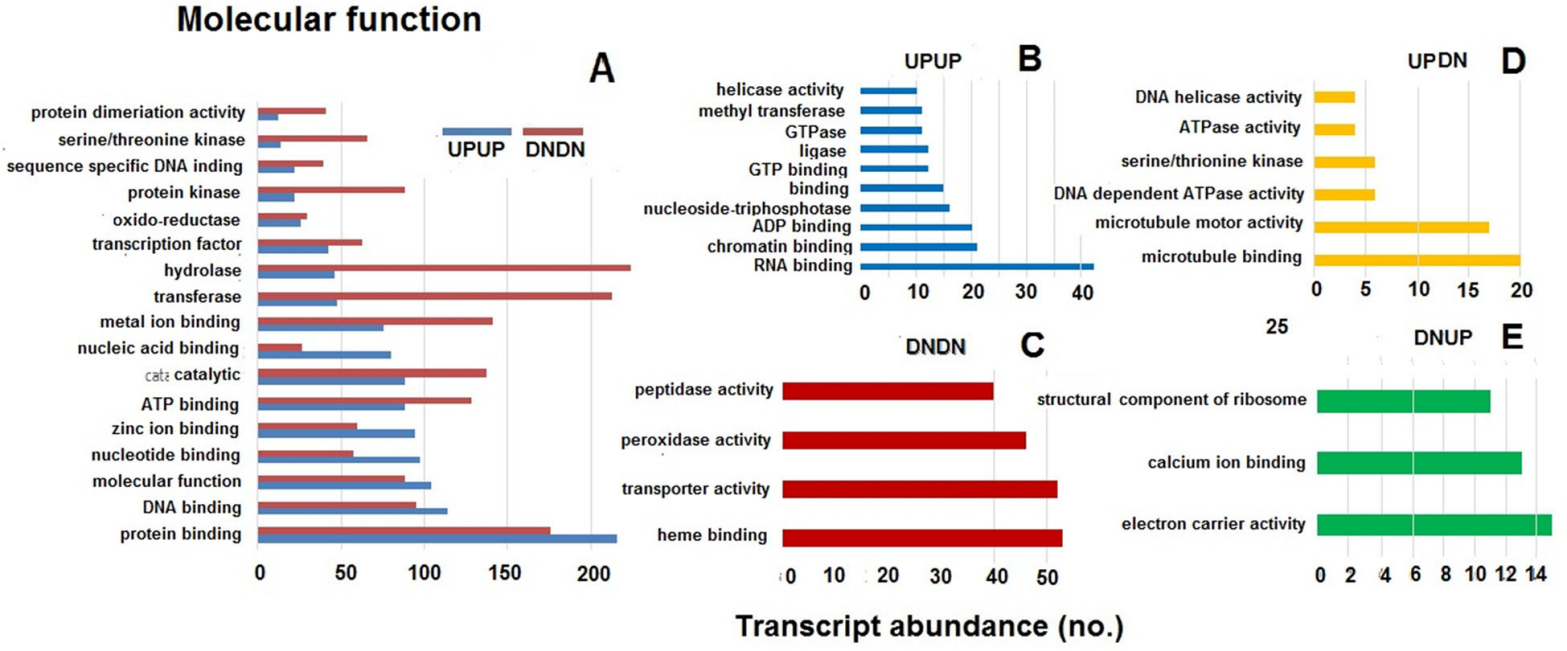
Abundance of transcripts of pathways under molecular function in differential expression groups in TR3RR plants infested with either BPH or WBPH. UPUP – upregulated against both BPH and WBPH; DNDN – down regulated against both BPH and WBPH; DNUP – down regulated against BPH but upregulated against WBPH; UPDN- upregulated against BPH but down regulated against WBPH

Gene Ontology (GO) enrichment was performed for all the DEGs in leaf sheath tissues of TR3RR plants when challenged by either BPH or WBPH. The DEGs were first grouped into UPUP, DNDN, UPDN and DNUP categories based on their expression (whether up or down regulated) against infestation by BPH or WBPH. The DEGs were further categorized into three major categories viz. biological process (BP), cellular component (CC) and molecular function (MF). However, several of the DEGs were annotated in two or all the three categories. Among the DEGs classified under biological process, 16 clusters were represented in both UPUP and DNDN groups; 6 clusters were exclusive to UPUP while 14 clusters were represented only in DNDN group (Fig. 4a, b, c). While 12 clusters belonged to the UPDN group, only three were in DNUP group (Fig. 4d, e). There were a higher number of transcripts commonly down regulated compared with the number of commonly upregulated transcript in several of the clusters except under the broad biological process cluster category. Though 12 clusters belonging to the UPDN group related to cell cycle, regulation of cell cycle related biological processes category, these transcripts were observed to be of low abundance. The three clusters under DNUP group were related to photosynthesis and primary metabolism. Under cellular component, 14 clusters of transcripts were common under UPUP and DNDN group (Fig. 5a). Five of these were exclusively upregulated against both the planthoppers, while six of clusters were exclusively down-regulated (Fig. 5b, c). Nine of the clusters categorized under the UPDN group were cell division-associated while five of the clusters under DNUP were associated with chloroplast and photosystem II (Fig. 5d, e). Under the molecular function category also (Fig. 6), more number of the transcripts were down regulated in 11 of the 17 of the clusters represented in both UPUP and DNDN groups (Fig. 6a). Of this category, 10 and 4 clusters were exclusive to UPUP and DNDN groups, respectively (Fig. 6b, c). Six clusters were in UPDN and three in DNUP groups. Genes in clusters relating to peroxidase, peptidase and transporter activity were down regulated against both BPH and WBPH. In contrast, genes pertaining to DNA helicase, ATPase, serine threonine kinase, and microtubular motor activities were upregulated against BPH but down-regulated against WBPH, and structural constituents of ribosome, calcium ion binding and electron carrier activity related genes were down-regulated against BPH but upregulated against WBPH. Structural constituents of ribosomes mostly represented the SSU-rRNA of endophytic bacterial genomes. As observed for the other two GO categories, unique transcripts associated with either of the planthopper’s challenge were low in abundance.

### Identification of miRNA Profiles

To identify the miRNAs, present in the RNA-seq data, all the transcript sequences in the size range of 20 to 24 nt were blasted against the miRbase. In all, 180,887 transcripts could be aligned with known rice miRNAs representing 713 families. Based on relative abundance of miRNA transcripts in plants subjected to infestation with either BPH or WBPH, 27 miRNAs were short-listed. These have been recognized to have 121 target genes. Of these, expression of 37 genes was observed to be modulated in the plants following planthopper feeding and these were identified to be modulated by 14 miRNAs (Table 3). Of the 37 genes targeted by 14 miRNAs (Supplementary Table S6), five genes were observed to have ≥2-fold higher expression against BPH with no change in expression level against WBPH. Two of these genes are associated with regulation of DNA replication and proteasome assembly. One of the genes (BGIOSGA006222 - LOC_Os02g35900 - thioredoxin, putative) displayed > 2-fold down-regulation against BPH with no change against WBPH. On the other hand, four of the genes had ≥2-fold increased expression against WBPH with no change against BPH. These genes were identified as thylakoid formation1- chloroplast precursor, CBS domain containing protein; phosphoglycerate mutase and a gene of unknown function, BGIOSGA023232. In addition, of the remaining two genes (BGIOSGA027084 and BGIOSGA011796), one is proposed to be a patatin, recorded ≥2-fold down-regulation against WBPH with no change against BPH. Thus, it is evident from the current results that miRNAs are targeting different sets of genes depending on whether the plants were challenged by BPH or WBPH.

**Table 3:** Changes in expression of selected miRNA targets in RIL TR3RR upon infestation with BPH or WBPH

miRNA ID	Target gene transcript ID	Fold change value for		MSU Loc ID	Description
		BPH	WBPH		
miR399k	BGIOSGA031778	2.5	NC	?	
	BGIOSGA010321	2.1	NC	LOC_Os03g39740	Regulation of DNA replication
miR399g	BGIOSGA002845	2.2	NC	LOC_Os01g07100	Transport, proteasome assembly
miR393a	BGIOSGA030203	2.1	NC	?	
	BGIOSGA016952	2.3	NC	?	
miR7692-5p	BGIOSGA006222	−2.2	NC	LOC_Os02g35900	thioredoxin, putative,
miR2869	BGIOSGA023232	NC	2.1	?	
miR5791	BGIOSGA030810	NC	2.0	LOC_Os09g26190	CBS domain containing protein
	BGIOSGA004148	NC	2.3	LOC_Os01g47190	phosphoglycerate mutase, putative
miR413	BGIOSGA024095	NC	2.1	LOC_Os07g37250	thylakoid formation1, chloroplast precursor
miR6245	BGIOSGA011796	NC	−2.1	? – Patatin	
miR413	BGIOSGA27084	NC	−2.2	?	

? Not listed in MSU Loc ID

*NC* No change in expression level

### Validation of DEGs through Reverse Transcription PCR (RT-PCR)

**Fig. 7:**
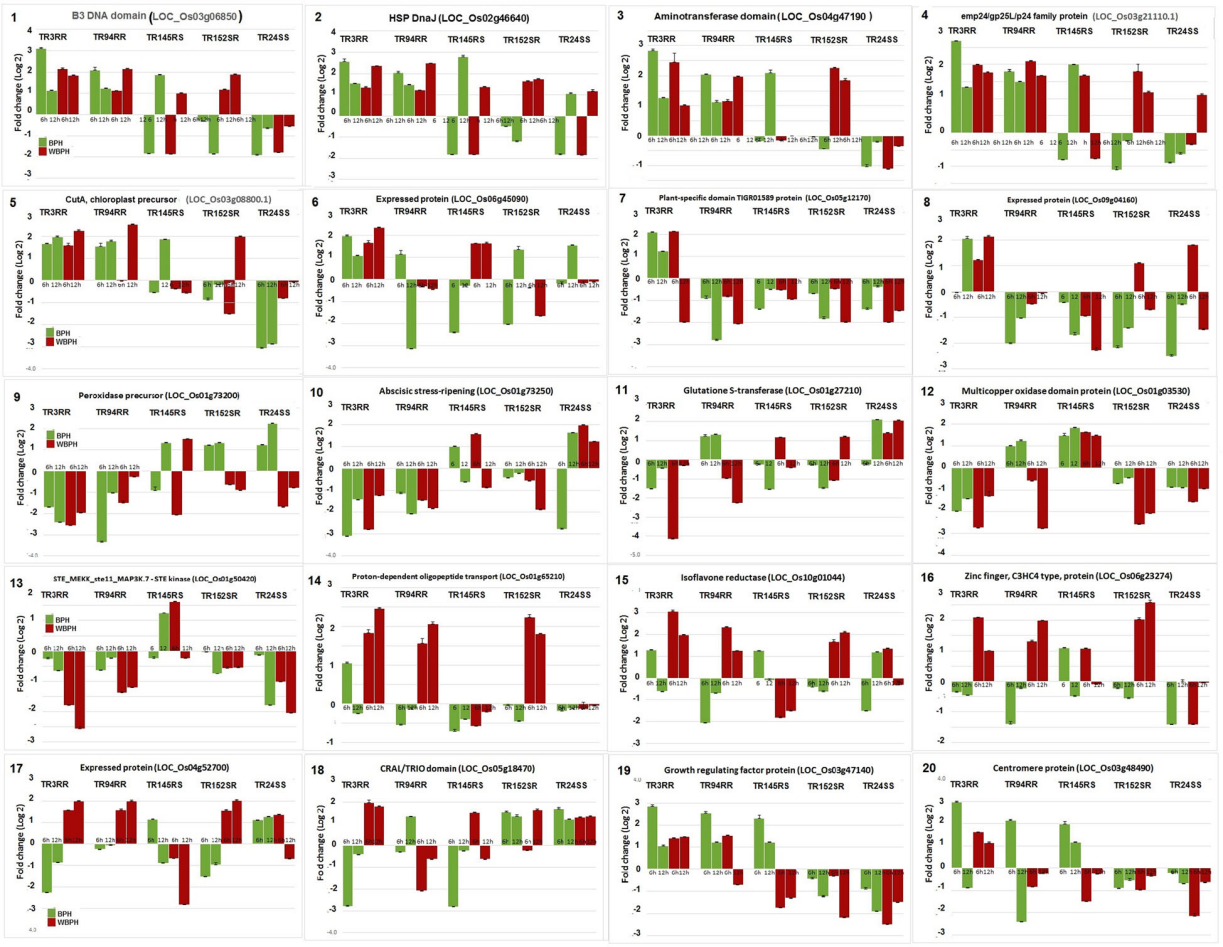
Relative expression of selected 20 genes, using qRT-PCR, in five RILs challenged with either BPH or WBPH. Bars indicate mean ± SD. Labels on the top indicate rice lines. Gene names with LOC numbers are provided

**Fig. 8:**
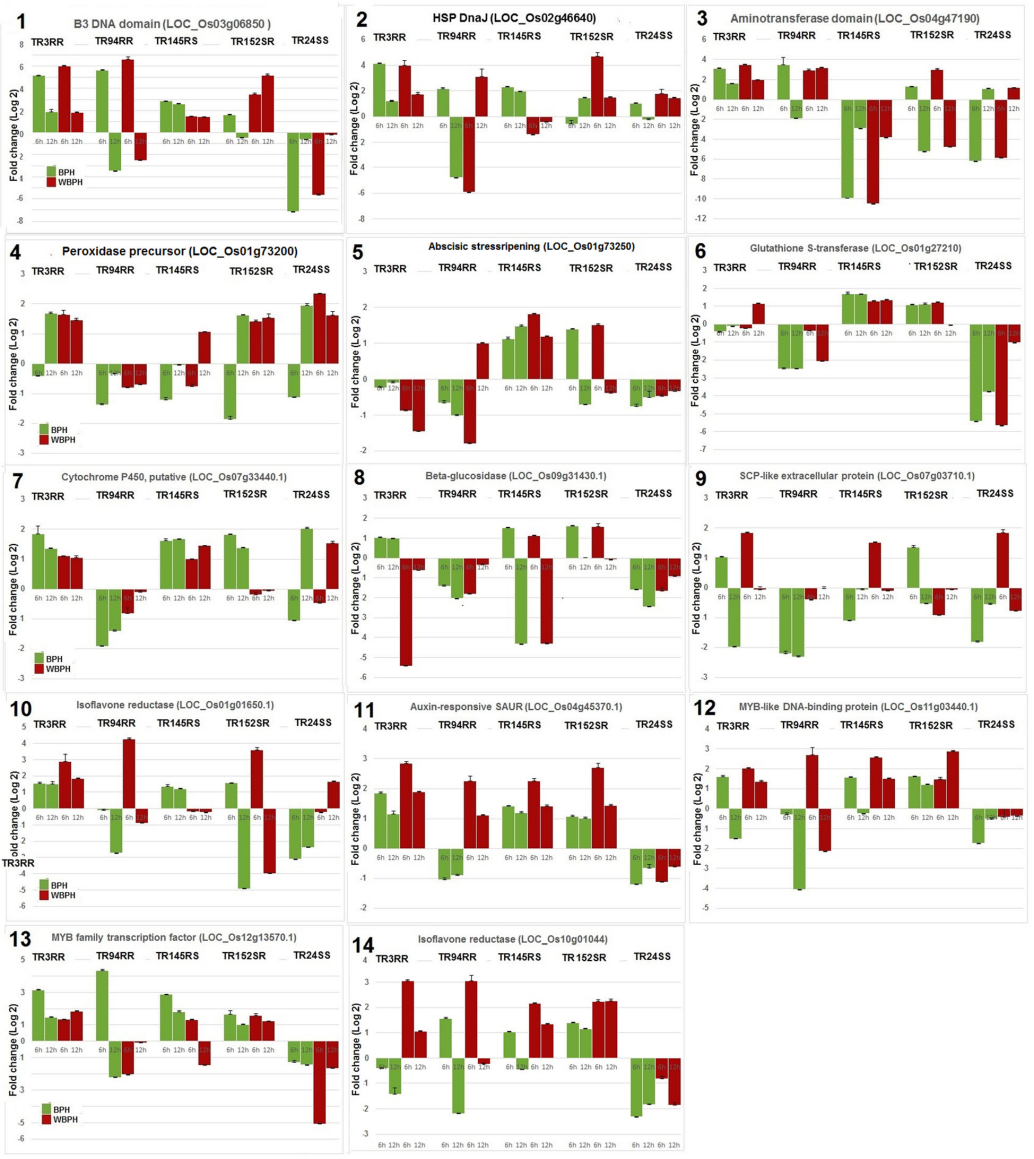
Relative expression of 14 selected genes, using real time qRT-PCR, in five RILs challenged with either BPH or WBPH. Bars indicate mean ± SD. Labels on the top indicate rice lines. Gene names with LOC numbers are provided

In all, 27 DEGs were validated in five of the RILs following infestation with either BPH or WBPH (Table 2). In the first set, 20 genes were analyzed through semi-quantitative RT-PCR (Fig. 7). In the second set, 14 genes including seven covered in the first set were validated through real time qRT-PCR in these genotypes (Fig. 8). Five of the RILs selected for the study included TR3RR, TR94RR, TR145RS, TR152SR and TR24SS based on their performance against the two planthoppers (Fig. 2). Expression of the target gene was monitored at two time points: 6 and 12 hai.

Of the 20 genes of the first set, 8 genes were selected from the UPUP group of DEGs (Fig. 7, panels 1 to 8); five genes each were from DNDN (Fig. 7, panels 9 to 13) and DNUP groups (Fig. 7, panels 14 to 18); and two genes from the UPDN group (Fig. 7, panels 19 & 20). The real-time PCR results were generally in agreement with RNA-seq data. Of the 8 genes in UPUP group, five genes (B3 DNA binding domain containing protein, HSP DnaJ protein-putative, aminotransferase domain containing protein, emp24/gp25L/p24 family protein and CutA, chloroplast precursor-putative) displayed distinct upregulation (≥ 2 fold) in both TR3RR and TR94RR against both BPH and WBPH; against BPH in TR145RS and against WBPH in TR152SR at one or both the time points (Fig. 7, panels 1 to 5). A similar trend was not distinctly observed for the remaining three genes (two undefined protein expressing genes on chromosomes 6 & 9 of rice and a plant-specific domain TIGR01589 containing protein coding gene), though upregulation was noted in TR3RR line against both BPH and WBPH.

All the five genes from the DNDN group displayed either down regulation or low levels (< 2-fold) of induction in four of the RILs against both BPH and WBPH. Interestingly, two of the genes (abscisic stress and ripening, and glutathione S-transferase registered significant induction (≥2- fold) in the susceptible TR24SS in 3 of 4 instances against both BPH and WBPH suggesting their possible role in susceptibility against the planthoppers. Five genes representing the DNUP group showed upregulation (≥ 2-fold) only against WBPH in three of the resistant test RILs (TR3RR, TR94RR and TR152SR), but not in the susceptible TR145RS and TR24SS. These genes displayed either down regulation or mild induction (< 2-fold) in all the RILs when infested with BPH. Only two genes (growth regulating factor protein and centromere protein coding genes) displayed threshold level of upregulation only against BPH in three of the resistant RILs (TR3RR, TR94RR and TR145RS). Thus, it was evident that despite 13 of the genes showing common response against BPH and WBPH with either induction or suppression, seven of the genes displayed differential response against BPH as versus WBPH.

Fourteen of the DEGs, as revealed by the RNA-seq analysis, were re-validated through quantitative real time RT-PCR. Three genes from UPUP group were re-analysed. Two of the genes (B3 DNA binding domain and heat shock protein DnaJ) showed similar expression as that observed in the first set (Fig. 8, panel 1 & 2). The third gene, aminotransferase domain containing protein did not show induction in one of the RILs, TR145RS, against BPH (Fig. 8, panel 3). Thus, these three genes represented common resistance pathways in rice RP2068 against both BPH and WBPH. The next set of six genes represented DNDN response group against the planthoppers. Three of these on re-analysis displayed identical response pattern as that observed in the first analysis using RT-PCR with either downregulation or poor induction (< 2-fold) against both the planthoppers (Fig. 8, panels 4 to 6). Unlike in the first set, glutathione S-transferase did not show induction in the susceptible TR24SS. But peroxidase precursor gene displayed some instances of induction in this line as also observed in the first set of RT-PCR analyses. The other three genes of the group (cytochrome P450-putative, Os9bglu30 - beta-glucosidase and SCP-like extracellular protein) also displayed either down-regulation or poor induction in all the test RILs with some instances of induction in TR24SS (Fig. 8, panels 7 to 9). Response of one of the two isoflavone reductase genes (LOC_Os01g01650.1) was re-tested in DNUP group and the results obtained were similar to those observed in the first set. Two more genes tested under this group (OsSAUR19 - Auxin-responsive SAUR gene family member- expressed and MYB-like DNA-binding domain containing protein - putative) showed distinct induction against WBPH but low-level induction (< 2-fold) or down-regulation against BPH in the resistant RILs (Fig. 8, panels 10–12). Significantly, these two genes were also noted to be induced under compatible interaction between the susceptible TR142RS and WBPH suggesting induction to be not directly related to resistance among the lines tested. Only one of the genes tested (MYB family transcription factor- putative) displayed distinct up-regulation under incompatible interactions between resistant RILs (TR3RR, TR94RR and TR145RS) and BPH but not against WBPH. Overall results of RT-PCR further highlighted differential resistance mechanism in RP2068 against BPH and WBPH.

Further, pathway analysis of these key 27 genes under four groups revealed major processes involving two or more of them (Supplementary Fig. S2). Trehalose biosynthetic pathway and oxidation-reduction pathway were significantly upregulated in plants under attack by either of the planthoppers and these were mediated by three of the key genes. Proline transport, methylation and protein processing and transport process were enhanced by two of the key genes. In contrast, pathways responsible for unsaturated fatty acid and glucosinolate biosynthetic process, proteolysis, cytokinesis by cell plate formation, response to oxidative stress and induced systemic resistance were down-regulated through the involvement of expression of two to four of the eight key genes in DNDN group. Significantly, pathways related to defense against bacteria and fungi, response to UV, oxidation-reduction, photosynthesis, regulation of transcription, oligo-peptide transport were the principal processes up-regulated by two to four of the eight key genes (DNUP group) in plants following infestation with WBPH, but not BPH. One of the two isoflavone reductase genes (i.e. LOC_Os01g01650.1) was part of the pathway responsible for photosynthesis and expression of proteins. Only the MYB transcription factor family gene (LOC_Os12g13570.1) was involved exclusively in defense against BPH, but not against WBPH. Thus, it is obvious from the results presented here that RP2068-derived plants respond differently against the challenge by BPH and WBPH.

## Discussion

The insect pest complex of rice has representations from several feeding guilds, and these include defoliators, tissue borers, gall formers, phloem feeders and several others. Each of these categories is represented by multiple species which are largely sympatric, occurring together at the same time and place, with exception being the Asian and African gall midges (*Orseolia oryzae; O. oryzivora*); rice green leafhopper and green rice leafhoppers (*Nephottetix virescens*; *N. cincticeps*) which are parapatric, with discrete geographic distribution. Among the sympatric species, members differ in their host range adaptation. Monophagous yellow stem borer (*Scirpophaga incertulas*) coexists with oligophagous striped stem borer (*Chilo suppressalis*) and polyphagous pink stem borer (*Sesamia inferens*). Monophagous BPH shares space with oligophagous WBPH and polyphagous small brown planthopper- (*Laodelphax striatellus;* https://www.plantwise.org/KnowledgeBank). Many studies have been conducted to understand interspecific interactions that results in mutual survival of the sympatric species (Cheng et al. 2001; Horgan et al. 2020). However, focused studies on how rice plant deploys different defense strategies against these sympatric planthopper species are not available. Information gathered through such studies will have valuable implications in pest management.

There have been several studies on understanding morphological, anatomical, physiological, biochemical and molecular basis of insect resistance in rice (see Du et al. 2020). One approach for these studies is based on forward genetics - with genetic characterization of resistance (R) genes, map-based cloning and understanding the function of these cloned genes. The other approach is reverse genetics- with analysis of genome wide gene expression of a set of resistant and susceptible varieties with appropriate controls and then observing and following the response of key genes to insect infestation. However, these two approaches are yet to converge and provide a vivid and comprehensive understanding of the diverse facets of insect resistance. At the outset, it is clear now that resistance in plants against chewing and tissue feeders involves JA (jasmonic acid)-mediated molecular pathways as compared to SA (salicylic acid)-mediated resistance that is deployed against sap sucking and gall forming pests (Baldwin and Preston 1999; Howe and Jander 2008; Wu and Baldwin 2010; Bentur et al. 2016). In the current study, omics tools have been employed to investigate molecular pathways triggered or suppressed in rice genotypes when challenged by BPH and WBPH to identify differences and commonality in defense pathways in rice against these planthoppers.

Rice line RP2068-18-3-5 (RP2068), derived from the cross between an elite cultivar Swarnadhan and the land race Velluthachera, is resistant to gall midge, BPH and WBPH like the parent land race (Bentur et al. 2011). A mapping population consisting of advanced generation RILs from TN1 X RP2068 cross has been used to tag and map the gall midge resistance gene *gm3* (Sama et al. 2014) and a BPH resistance gene *Bph33* (Naik et al. 2018). Evaluation of this mapping population against both BPH and WBPH indicated that resistance to the two planthoppers is independent of each other (Table 1) and resistance level against WBPH is slightly lower than that against BPH (Fig. 1). Based on the performance in four phenotypic tests, five RILs with varying degrees of resistance against BPH and WBPH (Fig. 2) but with a high level of genetic similarity (Supplementary Table S1) were selected for further studies. RNA-seq technique was adopted to follow differentially expressed genes (DEGs) in one of the selected RILs TR3RR following infestation with either BPH or WBPH with RNA samples pooled for two time points 6 and 12 hai. Analysis of data revealed larger number of DEGs to be down-regulated as compared to the number of DEGs upregulated (Fig. 3). A similar trend was noted in BPH susceptible WT (Nipponbare) plants during early stage of BPH feeding (Tan et al. 2020). But in a transgenic line of the same cultivar with *Bph6* gene (BPH6G) this trend was reversed. This difference is attributed to switching off of primary metabolism related activities in the plant to switch on defense related secondary metabolism and energy partitioning between the two (Guo et al. 2018). Significantly, higher number of DEGs were down regulated (5540) during interaction with BPH than with WBPH (4402) in TR3RR plant suggesting lower metabolic stress load. Further, Gene Ontology (GO) analysis of the DEGs pooled under four groups (UPUP, DNDN, UPDN, DNUP) also showed a predominance of down-regulated transcripts (Figs. 4a, 5a, 6a). Significantly, a small number of GO clusters represented differential response of the plant to the two planthopper infestations. While cell cycle, DNA repair related clusters were upregulated only against BPH, photosynthesis, protein synthesis and transport related genes were up-regulated only against WBPH under three GO groups (Figs. 4, 5). Thus, it appears that the plant is responding to BPH infestation at a higher order of resistance reaction where it is attempting to shut down several of the metabolic pathways compared with the situation when it is infested with WBPH where far fewer genes are down-regulated (Fig. 3) and in a manner more akin to a compensatory or tolerance mode of resistance reaction.

The role of micro RNA (miRNA) in modulation of BPH resistance in rice has become the focus of recent studies (Dai et al. 2019; Tan et al. 2020). Negative control of a growth regulation factor gene (*OsGRF8*) by OsmiR396 resulting in reduced expression of a key gene, *OsF3H*, in the flavonoid pathway led to BPH susceptibility in rice ZH11 (Dai et al. 2019). In the current study, two of the five DEGs selectively upregulated in TR3RR against BPH, modulated respectively by OsmiR399k and OsmiR399g, were involved in DNA regulation and replication, and in transport and proteasome assembly. Of the four target genes selectively upregulated against WBPH two were modulated by OsmiR5791. The CBS domain containing gene *Os09g26190* is reported to be associated with zinc deficiency tolerance and is a member of the network of genes related to photosynthesis, chlorophyll biosynthesis and proteins of thylakoid membrane (Lee et al. 2018). Another related gene *Os07g37250* was modulated by OsmiR413. Thus, emphasis here against WBPH is on enhancement in photosynthesis to compensate for the loss through insect feeding. The only gene selectively down-regulated against BPH and modulated by OsmiR7692-5p was coding a thioredoxin, putative, representing a major protein in phloem sap (Ishiwatari et al. 1995) on which planthoppers feed. Similarly, two uncharacterized genes were selectively down-regulated against WBPH and modulated by OsmiR6345 and OsmiR413. One of these is suggested to code for Patatin, a member of phosphor lipase A family and considered inhibitory for insect development and growth (Strickland et al. 1995). These three uniquely down-regulated genes suggest their negative role in expression of plant resistance; enhancing antibiosis effects against BPH through lowering levels of thioredoxin in phloem sap; and suppressing such effects against WBPH through lowering levels of Patatin. Importantly, the diversity in resistance mechanisms against the two planthoppers, is obvious as evidenced by the likely different roles of miRNAs play in gene regulation during the interaction of rice and these pests.

Based on the validation (using qRT-PCR) of expression of 27 shortlisted genes under four groups (UPUP, DNDN, UPDN, DNUP) three genes were identified that were upregulated against both the hoppers (Table 2, Figs. 7 and 8) including a B3 DNA binding domain containing protein coding gene (LOC_Os03g06850). Of the several cloned BPH R genes, *BPH29* (LOC_Os06g01860), codes for a B3 domain protein (Wang et al. 2015). Resistance conferred by this gene has been shown to arise due to loss of function. In contrast, LOC_Os03g06850 was significantly upregulated in the resistant RILs against both BPH and WBPH. Heat shock proteins (HSPs) are a family of proteins produced in response to stress, some of them like DnaJ act as chaperons. HSP genes have been often implicated in BPH resistance in rice (Wang et al. 2005; Wei et al. 2009; Naik et al. 2018). In the present study another HSP DnaJ gene responded against both the planthoppers. Other commonly upregulated genes included aminotransferase domain, emp24/gp25L/p24 protein associated with Golgi function; CUTA protein associated with chloroplast; plant specific TIGR01589 domain protein and two undefined expressed proteins. All these genes have not been earlier reported to be involved in plant defense against insects or pathogens.

Among the members of the DNDN group, two genes cytochrome P450-putative and Peroxidase precursor displayed unique expression profiles in that both were down-regulated in resistant RILs while these were up-regulated in susceptible RILs against both the planthoppers. The rice genome is rich in members of the cytochrome P450 super family (of 326 genes) with diverse regulatory role (Wei and Chen 2018), and some of which are implicated in BPH resistance in rice (Wei et al. 2009; Tan et al. 2020). An increased activity of P450 74A2 gene was noted in both compatible and incompatible interactions of resistant and susceptible NILs with *BPH15* gene (Wei et al. 2009). Of the two cytochrome P450 genes, one was found to be induced during early BPH feeding in transgenic line BPH6G while the other was repressed (Tan et al. 2020). Three genes representing peroxidase 12 precursor and two of peroxidase 2 precursor were found up-regulated during both compatible and incompatible interactions (Wei et al. 2009). But the magnitude of induction was more in compatible interaction. Peroxidase precursor gene (Os01g73200) noted in the present study was found to be induced during compatible interaction (Fig. 8). Four of the five POX genes were induced in susceptible lines against BPH (Wei et al. 2009). These genes are involved in Jasmonic Acid (JA) biosynthesis. Induction of JA pathway genes during compatible interaction by the planthoppers may be a counter defense strategy to suppress salicylic acid (SA) pathway defense as reported for the pathogenic fungi (Okada et al. 2015) and bacteria (Nomura et al. 2005). Glutathione S-transferase is another large family in rice with 59 genes that have detoxifying function against xenobiotics (Soranzo et al. 2004). Down-regulation of two members of this gene family was noted in rice lines with or without *Bph15* gene during both compatible and incompatible interactions (Wei et al. 2009). Suppression of this gene is likely to represent another counter defense strategy employed by the planthoppers. Wang et al. (2005) noted up-regulation of a member of 21-gene family of beta glucosidases during resistance reaction in rice lines B5 (with *Bph14* and *Bph15*genes) and they speculated that this gene may be involved in functions other than volatile emission. MAP 3kinase gene family, of which STE_MEKK_ste11_MAP3K.7 - STE kinase is a member, is reported to be involved in rice resistance to BPH with down-regulation of one of the members being reported by Wang et al. (2005). Other genes in this DNDN category i.e. multicopper oxidase domain is reported to be involved in conferring blast resistance in silicon-amended rice (Brunings et al. 2009) while SCP like extra cellular protein is not reported to be involved in rice defense against biotic stresses. Abscisic acid stress and ripening (*ASR*) gene is a member of the transcription factor family which altered expression of *OsASR2*, another member of the family, and influenced rice resistance to bacterial blight and sheath blight (Li et al. 2018).

Significantly, genes belonging to the groups UPDN and DNUP revealed differential mechanisms. Two isoflavone reductase-like (IRL) genes on chromosome 1 and 10 were dramatically induced in resistant RILs only against WBPH and not against BPH. IRLs of rice are not true isoflavone reductases like those of legumes and do not produce natural isoflavonoid products by acting on the substrate 2-hydroxy isoflavonoids (Kim et al. 2003). Nonetheless, IRLs are developmentally regulated or induced by biotic or abiotic stresses such as rice blast. Auxin-responsive SAURs, a 58-member RNA gene family in rice (Jain et al. 2006), plays a role in auxin synthesis and transport (Xu et al. 2017). The observation that high level of induction of *OsSAUR19* in plants against WBPH, but not against BPH, may suggest suppression of auxin pathways in the present study against BPH infestation. C3HC4-type zinc finger proteins represent one of the largest groups of transcription factors in plants involved in stress responses. A member of Dof zinc finger family of transcription factors was differentially down-regulated following BPH infestation (Yuan et al. 2005; Wang et al. 2005) while another member - zinc finger protein gene was upregulated (Li et al. 2016). In the present study, LOC_Os06g23274 coding for Zinc finger, C3HC4, domain protein was differentially expressed displaying its selective role against WBPH. The other two genes in this group: proton-dependent oligopeptide transporter and CRAL/TRIO domain protein, besides an undefined expressed protein, have not been earlier reported to be involved in plant defense against insects or pathogens. Interestingly, two of the MYB transcription factor representing genes showed reciprocal response to planthopper infestation. While Os12g13570.1 was up-regulated against BPH and down-regulated against WBPH, Os11g03440.1 with MYB-like DNA-binding domain recorded down-regulation against BPH and up-regulation against WBPH. The role of various transcription factors in expression of BPH resistance in the rice variety Rathu Heenati has been studied (Wang et al. 2012). In this study, most members of MYB family were observed to be down regulated after BPH infestation, suggesting these to be related to reduced photosynthesis rate, stomatal conductance and transpiration rate. Another two genes coding for a growth regulatory factor and a centromere protein were also observed to be up-regulated against BPH and these were down-regulated against WBPH. Dai et al. (2019) showed the link between miR396 and a growth regulating factor gene *OsGRF8* and a gene, *OsF3H*, involved in the flavonoid biosynthetic pathway, to conclude that miR396 has negative control of BPH resistance in the susceptible genotype. Likewise, the growth regulatory factor gene, LOC_Os03g47140, in the present study is being targeted by miR396f (Wen et al. 2016) or miR396c-3p (Tan et al. 2020). However, transcripts of all the members of this family of miR396 were more abundant in BPH-challenged plants than in WBPH-challenged plants. The centromere protein gene has not been reported associated with biotic stresses in plants as has been observed in this study. Pathway analysis with the 27 key genes (Supplementary Fig. S2) further supported the facts that the response of RP2068 against WBPH was more compensatory or tolerance in nature with increased photosynthesis and protein synthesis and transport in the affected tissue while its response against BPH involved the induction of several active defense pathways targeting antibiosis.

## Conclusion

RNA-seq data generated from infested and control tissues of TR3RR – a RIL derived from a cross between susceptible TN1 rice and resistant RP2068 challenged with BPH or WBPH – helped to identify the pathway genes involved in resistance. Our results revealed that a larger number of DEGs were down-regulated, in comparison to up-regulated DEGs, in plants following the planthopper infestation. Identification of unique clusters of GO groups responding exclusively to one of the hoppers suggested diversity in defense strategies adopted by the plant against two different planthoppers from the same feeding guild. Further, functional validation of the selected 27 genes showed unique role of genes such as IRL, a growth regulating factor (GRF) and two members of MYB transcription factor family against one of the planthoppers. This, to the best of our knowledge, is the first study to demonstrate the selective expression of rice host genes upon attack from two major insect pests of rice using genetically similar host material. Such studies are not only important to dissect the plant responses to different insect pests but information derived from such studies are urgently required to consciously combine relevant resistance strategies against both the planthoppers for their effective management.

## Materials and Methods

### Insects

Adults of both BPH and WBPH were collected from farmers’ field in Nalgonda district of Telangana state, India, during 2014–2015 and separate colonies were established on TN1 rice in greenhouses at ABF, Hyderabad. Care was taken to prevent population admixture. Nymphs or adults, arising from these populations reared in the greenhouse, of specified age/stage were used for the experiments.

### Screening and Selection of Recombinant Inbred Lines (RILs)

A previously developed mapping population (F_14_; 180 lines) (Sama et al. 2014) derived from a cross between rice varieties TN1 and RP2068 (TR) was used in the current study. All the 180 RILs were subjected to standard seedbox screening test (SSST), nymphal survival (NS), nymphal preference (NP) and days to wilt (DW) separately against BPH and WBPH.

### Standard Seedbox Screening Test (SSST)

Degree of resistance, in terms of damage score, of parents and F_14_ RILs was measured in standard seedbox screening test (SSST). In this method the parents and test lines were infested with 2nd instar BPH/WBPH nymphs, on an average of 8–10 nymphs per seedling 10–12 days after sowing (Naik et al. 2018). The test lines were arranged in a randomized complete block design (RCBD) and replicated three times. Susceptible TN1 was sown in two rows at the edge of box on both the sides while the resistant check (PTB33 for BPH and MO1 for WBPH) was sown in one row in the center. These plants were observed for damage and scored as per the standard evaluation system (SES) for rice (IRRI 2013) on a scale 0 to 9 when 90% of TN1 on both the rows were dead in about 8 to 12 days after insect release. Each test entry was scored by recording damage to each seedling; subsequently, the score for each replication was averaged and then the grand mean of the three replications was derived for the entry. Entries with damage score 1 to 3 against BPH and with score 1 to 4 against WBPH for rice were considered as resistant (as per SES) while those with score ≥ 8.0 were treated as susceptible for this study (Fig. 1).

### Nymphal Survival (NS)

Nymphal survival was recorded on 30-day-old potted test plants of the RILs along with the resistant RP2068 and the susceptible TN1 parents, and the resistant checks PTB33 (BPH) or MO1 (WBPH). The plants were raised in 500 ml plastic pots with puddled soil from rice field which were randomized (RCBD) and then infested with ten 1st or 2nd instar nymphs per plant covered with a mylar film tube cage. Survival of the insects on the plants was observed daily and number of surviving nymphs was recorded until all surviving nymphs metamorphosed into adults. Three replications were maintained. Nymphal survival was expressed as percentage and means. Entries with ≤60% survival against BPH and WBPH were considered as resistant while those entries with > 60% survival were described as susceptible. For statistical comparison of means, values were transformed into arc-sine values (Gomez and Gomez 1984).

### Days to Wilt (DW)

The tolerance component of resistance was studied using days to wilt test (Geethanjali et al. 2009). Briefly, the test plants along with the parents and the resistant checks were grown singly in 500 ml plastic pots. When plants were 30 days old, pots were randomized and covered with mylar tube cages and infested with 50 1st or 2nd instar nymphs of BPH/WBPH. Plants were observed daily. The day on which plant wilted completely was recorded. One pot represented a replication and the test was replicated three times. Entries with plants surviving more than 10 days were considered as resistant and other entries were treated as susceptible.

### Nymphal Preferences

Antixenosis or non-preference (of nymphs for settling on seedlings) type of resistance mechanism was assessed while conducting the standard seed box screening test (Heinrichs et al. 1985). Planting of seedlings was conducted in a similar way describe in SSST above. After 10-12 days of sowing, second instar nymphs of BPH/WBPH were released on the seedlings on an average of 8–10 nymphs per seedling. Each seed box was covered with a mylar film cage and was treated as a replication. Number of nymphs on each seedling was counted 24 and 48 h after infestation. Percentage of insects settled on each of the test entry was computed based on total number of insects noted for each replication (box). Entries with ≤10% of nymphs settling on plants were considered as resistant and those with > 10% of nymphs as susceptible. For statistical comparison of means, values were transformed into arc-sine values (Gomez and Gomez 1984). One-way ANOVA was performed on data of each phenotypic test and means were separated by HSD following Tukey and Kramer method (Tukey 1953) on MS Excel (https://www.youtube.com/watch?v=N7mkI8_xxc4&feature=emb_logo).

Based on these tests, RILs showing RR (resistant to BPH and WBPH), RS (resistant to BPH only), SR (resistant to WBPH only) and SS (susceptible to both BPH and WBPH) were identified (Table 1). From these, five RILs with genetic similarity of ≥40%, based on screening of these RILs using 137 polymorphic markers (Sama et al. 2014; Naik et al. 2018; Sahu et al. unpublished), were chosen for the study (Supplementary Table S1). Performance of the selected RILs in each of the tests was compared through paired t-test with equal variance and one tailed distribution options (MS Excel, Office 365).

### Sample Collection for RNA-Seq

In order to identify the pathway genes responsible for conferring resistance to BPH and WBPH in RP2068, an NGS protocol was used. RNA sequencing (RNA-seq), was conducted with RIL TR3RR (resistant to both BPH & WBPH). TR3RR seedlings were raised in nine 3 L plastic bucket pots (6 seedlings/pot; 3 pots as uninfested control, 3 pots infested with BPH and 3 pots infested with WBPH). Fifteen days after sowing, the designated pots were infested with 1st-2nd instar nymphs (5 nymphs for BPH and 10 nymphs for WBPH/plant). Lower leaf sheath samples (from three individual plants/pot) were collected for 3 biological replications at two different time points: 6 and 12 h after infestation (hai). Total RNA was isolated from these nine samples, including uninfested control plants, using RNeasy plant mini kit (Qiagen, Germany) as per the manufacturer’s guidelines. The RNA samples collected for each of the two time points (6 hai and 12 hai) for each replication were pooled prior to sequencing. RNA sequencing was carried out by M/s Genotypic Technology Pvt. Ltd., Bengaluru, India.

### RNA Quality Control

The concentration and purity of the RNA was evaluated using the Nanodrop Spectrophotometer (Thermo Scientific 2000). The integrity of the extracted RNA was analyzed on the Agilent Bioanalyzer 2200 (Agilent, CA, USA) using the manufacturer’s protocols.

### qRT-PCR

For validation studies using qRT-PCR, similar experiment setup was repeated and in addition to TR3RR, four additional RILs (TR94RR, TR145RS, TR152SR and TR24SS) were included. Seedlings were raised in 3 L plastic bucket pots and 15 days after sowing they were infested with 1st-2nd instar nymphs of BPH or WBPH. Leaf sheath samples were collected from 3 replications of control and infested plants at two different time points: 6 and 12 hai. RNA isolation and cDNA synthesis were carried out following standard protocols (Biorad, USA). Twenty-seven genes were selected and validated; first 20 genes using semi-quantitative RT-PCR and 14 genes (including seven of the earlier set) were selected for real time quantitative RT-PCR (Table 2). Of the 27 genes selected for validation, eight genes each were from UPUP (noted to be upregulated during both BPH and WBPH infestation), DNDN (down regulated against both the planthoppers) and DNUP groups while three genes were from UPDN group, as revealed by the RNA-seq data (Table 2).

### Library Preparation and Sequencing

RNA sequencing libraries were prepared with Illumina-compatible NEBNext Ultra Directional RNA Library Prep Kit for Illumina (New England BioLabs, MA, USA) at M/s Genotypic Technology Pvt. Ltd., Bengaluru, India.

One μg of total RNA was taken for mRNA isolation, fragmentation and priming. Fragmented and primed mRNA was further subjected to first strand cDNA synthesis in the presence of Actinomycin D (Gibco, life technologies, CA, USA) followed by second strand synthesis. The double stranded cDNA was purified using HighPrep magnetic beads (Magbio Genomics Inc., USA). Purified double-stranded cDNA was end-repaired, adenylated and ligated to Illumina multiplex barcode adapters as per manufacturer’s protocol.

Illumina Universal Adapters were used in the study. Adapter-ligated cDNA was purified using HighPrep magnetic beads and was subjected to 14 cycles of Indexing-PCR (37 °C for 15mins followed by denaturation at 98 °C for 30 s, cycling (98 °C for 10s, 65 °C for 75 s) and 65 °C for 5mins) to enrich the adapter-ligated fragments. The final PCR product (sequencing library) was purified with HighPrep magnetic beads, followed by library quality control check. Illumina-compatible sequencing libraries were quantified by Qubit fluorometer (Thermo Fisher Scientific, MA, USA) and its fragment size distribution were analyzed on Agilent 2200 Tape station. The libraries were sequenced by using Illumina HiSeq 4000 sequencer (Illumina, San Diego, USA) for 2 X 150 paired-end chemistry following manufacturer’s procedure.

### Tool Description

Analysis pipeline and the different software used for analyzing the raw sequencing data obtained from the HiSeq sequencer were as follows:

### Quality Check

The raw data generated was quality-checked using FastQC. Reads were preprocessed to remove the adapter sequences and removal of the low-quality bases (<q30). Pre-processing of the data was done with Cutadapt (Martin 2011). HISAT-2, which is a splice aligner, was used to align the high-quality data to the reference genome with the default parameters (Kim et al. 2015).

### Transcript Abundance Estimate

Cufflinks4 was used to estimate and calculate transcript abundance (Trapnell et al. 2010). The output from the analysis resulted in normalized read counts in the form of FPKM values. FPKM is a unit of measuring gene/transcript expression (Fragments Per Kilobase of transcript per Million mapped reads).

### Genome Mapping

All the processed reads were aligned to the *Oryza sativa indica* genome downloaded from the EnsemblPlants database. An average of 44.93% of the reads were aligned to the reference genome. The alignment (BAM) files were viewed and inspected in standard genome viewer IGV browser (Thorvaldsdóttir et al. 2013).

### Transcript Identification and Quantification

Transcripts were identified and quantified based on aligned reads. Transcript expression were generated through cufflinks. On an average 23,466 transcripts were expressed across all samples. Compiled expression profile at transcript level has been represented in form of FPKM Matrix [GT_SO_8261_Read_Count_Matrix.xlsx or Table S2].

### Transcript Assembly

Cufflinks-2.2.1 was used to assemble transcripts, estimate their abundances, and test for differential expression and regulation in RNA-Seq samples and to estimate the relative abundance of these transcripts based on read distribution support while accounting for biases in library preparation protocols (Trapnell et al. 2010).

After mapping the sequences to the reference genome, the mapped files, as provided by Cufflink-2.2.1 software, was used to generate a transcriptome assembly. These assemblies were merged using the Cuffmerge, option which is included with in the Cufflinks package (Trapnell et al. 2010). The resulting alignment (in BAM file format) was used to generate transcript annotations (GTF format) using default Cufflinks parameters. This merged assembly provided a uniform basis for calculating gene and transcript expression under each treatment. The merged assembly was next analysed by Cuffdiff 4 to calculate expression levels and assign statistical significance to observed changes in expression levels (Trapnell et al. 2012).

### Differential Expression Analysis

Cuffdiff4 was used to calculate the differentially expressed transcripts and categorize them into UP-, Down- and Neutrally regulated transcripts based on the log2fold change value at *P* ≤ 0.05 (Trapnell et al. 2012). Group-wise comparisons were performed to identify differentially regulated transcripts between two treatments. The transcripts that showed a log2fold change value less than − 1 were categorized as down regulated; those with greater than 1 were categorized as upregulated and ones with the log2fold change values between − 1 to 1 were categorized as neutrally regulated.

### Gene Ontology (GO) and Pathway Analysis

For each transcript, gene ontologies were downloaded from ensembl biomart database (https://www.ensembl.org/biomart/martview). These GO were mapped to the differentially expressed transcripts/genes (DEG). Next, pathways for each gene were obtained from multiple database such as KEGG pathways and biomart and the compiled pathways for each gene were mapped to the DEGs.

### MapMan

Functional annotation and metabolic pathway analysis were performed using MapMan (Thimm et al. 2004; Usadel et al. 2005). MapMan was also used to identify the functional categories associated with a set of sequences (e.g., differentially expressed) and to find the metabolic pathways or other cellular functions up- or down-regulated as revealed by the RNA-seq data. Functional classification in the mapping file (X4.2_Oryza_indica) that structures the rice gene from an TIGR into distinct metabolic and cellular processes from the MapMan program was used. Differentially expressed rice genes were functionally annotated by performing Basic Local Alignment Search Tool (BLAST) alignment against the TIGR database. MapMan software was employed to show the differences in gene expression in different cellular and metabolic process. Ratios were expressed in a log_2_ scale for importing into the software and changes in expression were displayed via a false color code.

### In Silico Analysis for miRNAs

The raw sequence data obtained for both control and infested samples (BPH and WBPH) were used as input for psRNATarget software (http://plantgrn.noble.org/psRNATarget/) to predict plant small RNA targets using default parameters. The miRNA precursors/miRNAs sequence of rice in the miRBase 21.0 (http://www.mirbase.org/search.shtml) database was used to identify the mature sequences and the families of miRNAs to which it belongs. The miRNA sequences were used as input in RNA fold database (http://rna.tbi.univie.ac.at/) to predict their secondary structure.

Mature rice miRNA sequences for these families was downloaded from miRbase (http://www.mirbase.org/cgi-bin/mirna_entry.pl) and the sequence and BLAST analyses (Ref) was performed against *indica* rice genome (http://plants.ensembl.org/Oryza_indica/Tools/Blast/). Genes listed as overlapping/homologous with the reference sequence were considered as the target genes. Net, expression profiles of these genes in our mRNA library were obtained and MSU *japonica* locus Ids and the putative nature and function of the genes were identified (http://rice.hzau.edu.cn/cgi-bin/rice2/id_mapping_rs2).

### In Silico Pathway Analysis

We performed network analysis using RiceNet v2 (Lee et al. 2018). All the 27 genes were queried separately in four groups (Table 2) under gene prioritization based on context associated hubs for discerning whether any of the major networks were invoked as defined by the representative genes in these four groups. Pathways involving two or more of the queried genes were identified.

### Semi-Quantitative PCR

The transcriptome analysis results were further validated via semi-quantitative reverse transcription polymerase chain reaction (RT-PCR) with two types of samples: (i) the same RNA samples that were used for RNA-seq analysis and (ii) newly isolated RNA samples from independent infestation. About 3 μg of RNA was used for first-strand cDNA synthesis using the iScript cDNA synthesis kit (Bio-Rad, USA) following the manufacturer’s guidelines. Gene-specific primers for the RT-PCR were designed using Primer3 Software (https://bioinfo.ut.ee/primer3-0.4.0) (Supplementay Table S3). The PCR mix contained 30 ng of cDNA, 0.5 μM primers (forward and reverse each), 200 μM each of the dNTPs, 1 Unit of Taq polymerase and Taq buffer (Bangalore Genei Pvt. Ltd., India). The optimum PCR conditions including cycle number and cDNA amounts were standardized for each gene separately. The PCR, products were run on 1.5% agarose gel at 90 V for 1 h. and the agarose gels were documented using the Alpha Imager EP system (Cell Biosciences, USA). The captured images were analyzed using ImageJ software (Schneider et al. 2012). Twenty genes were analyzed (Fig. 7) with rice ubiquitin (GenBank accession number AK059694) as a reference gene for normalization and the fold change values were calculated between the relative expression values (REVs) of infested and control plants. One-way ANOVA was performed on fold change value data for each gene against specific planthopper and time point and means were separated by HSD following Tukey and Kramer method (Tukey 1953) on MS Excel (https://www.youtube.com/watch?v=N7mkI8_xxc4&feature=emb_logo). Negative values were expressed as decimal fraction for analysis. Results are expressed as a graph constructed based on the log (2) values of the fold change using MS Excel (Supplementary Table S4, Fig. 7).

### Quantitative RT-PCR

Real time RT-PCR was performed using CFX96 Real Time PCR System with the SYBR green chemistry (Bio-Rad, USA) according to the manufacturer’s instructions. Rice ubiquitin gene, OsUbq (GenBank accession no. AK059694), was used as the endogenous control. Real Time PCR reaction volume of 10 μl contained 5 μl SYBR Green PCR Master Mix (Bio-Rad, USA), 500 nM each of forward and reverse primers and 30 ng of the cDNA samples. To calculate mean relative expression levels, cDNAs from three independent biological samples in two technical replications each were used. PCR was initiated with denaturation at 95 °C for 5 min followed by 40 cycles of denaturation at 95 °C for 10s and annealing and extension at 60 °C for 30s. After 40 cycles, a melt curve analysis was carried out to determine the specificity of the reaction. After normalization, quantity of each mRNA was calculated from the threshold points (CT) located in the log-linear range. The data from different PCR runs or cDNA samples were compared by using the mean of CT values of the three biological replicates that was normalized to the mean of CT values of the endogenous gene. The relative standard curve method was used for the quantification of mRNA levels and displayed as Relative Expression Values (REV). Expression ratios were calculated using the 2^–∆∆Ct^ method (Livak and Schmittgen 2001). The data were analyzed using the Bio-Rad CFX Manager 3.1 Software (Bio-Rad, USA) with default baseline and threshold. Relative transcription levels are presented graphically. In all expression of 14 identified genes were validated in leaf sheath tissues of the plants (exposed to BPH or WBPH), separately for each time point. Results are presented as mean ± SD of relative expression in comparison with corresponding uninfested control sample. One-way ANOVA was performed on fold change value data for each gene against specific planthopper and time point and means were separated by HSD following Tukey and Kramer method (Tukey 1953) on MS Excel (https://www.youtube.com/watch?v=N7mkI8_xxc4&feature=emb_logo). Negative values were expressed as decimal fraction for analysis. (Supplementary Table S5, Fig. 8).

### Data Availability

The sequence data (raw data) generated in this study has been deposited at NCBI Sequence Read Archive (SRA) database (www.ncbi.nlm.nih.gov/sra). The Bioproject accession number for Molecular basis of differential resistance in rice line RP2068-18-3-5 against BPH and WBPH is PRJNA577384, and SRA experimental accession numbers are SRR10394603, SRR10394602, SRR10394598, SRR10394601, SRR10394600, SRR10394597, SRR10394599, SRR10394596 and SRR10394595.

## Supplementary Information

**Additional file 1: Supplementary Data File No 1.** with list of DEGs in TR3RR RIL following BPH infestation

**Additional file 2: Supplementary Data File No 2.** with list of DEGs in TR3RR RIL following WBPH infestation

**Additional file 3: Supplementary Tables S1.** Genetic similarities among the selected TR RILs*. **Table S2.** Overview of raw reads and mapped sequences for all the nine samples. **Table S3.** Sequence information of primers used for qRT-PCR of the shortlisted DEGs identified from the RNA-seq data

**Additional file 4: Supplementary Table S4.** Mean fold values and Tukey’s HSD for separation of means for 20 genes validated with RT-PCR (semi-quantitative)

**Additional file 5: Supplementary Table S5.** Mean fold values and Tukey’s HSD for separation of means for 14 genes validated with qRT-PCR (quantitative)

**Additional file 6: Supplementary Table S6.** List of significant miRNA and their reported target genes in RILs induced by infestation either by BPH or WBPH

**Additional file 7: Supplementary Fig S1.** Scatter plot representing correlation coefficient between samples

**Additional file 8: Supplementary Fig S2**. Pathway analysis for key genes listed in Table 2

## Data Availability

Additional data files 1 and 2 submitted. The sequence data (raw data) generated in this study has been deposited at NCBI Sequence Read Archive (SRA) database (www.ncbi.nlm.nih.gov/sra). The Bioproject accession number for Molecular basis of differential resistance in rice line RP2068-18-3-5 against BPH and WBPH is PRJNA577384, and SRA experimental accession numbers are SRR10394603, SRR10394602, SRR10394598, SRR10394601, SRR10394600, SRR10394597, SRR10394599, SRR10394596 and SRR10394595.

## References

[CR1] Baldwin IT, Preston CA (1999). The eco-physiological complexity of plant responses to insect herbivores. Planta.

[CR2] Bentur JS, Padma Kumari AP, Jhansi Lakshmi V, Padmavathi C, Kondala Rao Y, Amudhan S, Pasalu IC (2011). Insect Resistance in Rice. Technical Bulletin #51 Directorate of Rice Research, Rajendranagar, Hyderabad, A P, India, 86.

[CR3] Bentur JS, Rawat N, Divya D, Sinha DK, Agarrwal R, Atray I, Nair S (2016). Rice–gall midge interactions: battle for survival. J Insect Physiol.

[CR4] Bentur JS, Viraktamath BC (2008). Rice planthoppers strike back. A report on second international rice conference on rice planthoppers held at IRRI, Philippines during 23-25 June. Curr Sci.

[CR5] Bottrell DG, Schoenly KG (2012). Resurrecting the ghost of green revolutions past: the brown planthopper as a recurring threat to high-yielding rice production in tropical Asia. J Asia Pac Entomol.

[CR6] Brar DS, Virk PS, Jena KK, Khush GS (2009). Breeding for resistance to planthoppers in rice. Plant hoppers: new threats to the sustainability of intensive rice production systems in Asia.

[CR7] Brunings AM, Datnoff LE, Ma JF, Mitani N, Nagamura Y, Rathinasabapathi B, Kirst M (2009). Differential gene expression of rice in response to silicon and rice blast fungus *Magnaporthe oryzae*. Ann Appl Biol.

[CR8] Cheng J, Zhao W, Lou Y, Zhu Z (2001). Intra-and inter-specific effects of the brown planthopper and white backed planthopper on their population performance. J Asia Pac Entomol.

[CR9] Dai Z, Tan J, Zhou C, Yang X, Yang F, Zhang S, Sun S, Miao X, Shi Z (2019). The OsmiR396–Os GRF 8–OsF3H-flavonoid pathway mediates resistance to the brown planthopper in rice (*Oryza sativa*). Plant Biotechnol J.

[CR10] Du B, Chen R, Guo J, He G (2020). Current understanding of the genomic, genetic, and molecular control of insect resistance in rice. Mol Breed.

[CR11] Fan F, Li N, Chen Y, Liu X, Sun H, Wang J, He G, Zhu Y, Li S (2017). Development of elite BPH-resistant wide-spectrum restorer lines for three and two line hybrid rice. Front Plant Sci.

[CR12] Fujita D, Kohli A, Horgan FG (2013). Rice resistance to planthoppers and leafhoppers. Crit Rev Plant Sci.

[CR13] Ge Y, Han J, Zhou G, Xu Y, Ding Y, Shi M, Guo C, Wu G (2018). Silencing of miR156 confers enhanced resistance to brown planthopper in rice. Planta.

[CR14] Geethanjali S, Kadirvel P, Gunathilagaraj K, Maheswaran M (2009). Detection of quantitative trait loci (QTL) associated with resistance to whitebacked planthopper (*Sogatella furcifera*) in rice (*Oryza sativa*). Plant Breed.

[CR15] Gomez KA , Gomez AA (1984) Statistical procedures for agricultural research, 2nd edition. USA: International Rice Research Institute and Wiley-Interscience

[CR16] Guo J, Xu C, Wu D, Zhao Y, Qiu Y, Wang X, Ouyang Y, Cai B, Liu X, Jing S, Shangguan X (2018). Bph6 encodes an exocyst-localized protein and confers broad resistance to planthoppers in rice. Nat Genet.

[CR17] Haliru BS, Rafii MY, Mazlan N, Ramlee SI, Muhammad I, Akos IS, Halidu J, Swaray S, Bashir YR (2020). Recent strategies for detection and improvement of brown planthopper resistance genes in rice: a review. Plants.

[CR18] Han Y, Wu C, Yang L, Zhang D, Xiao Y (2018). Resistance to *Nilaparvata lugens* in rice lines introgressed with the resistance genes *Bph14* and *Bph15* and related resistance types. PLoS One.

[CR19] Heinrichs EA, Medrano FG, Rapusas HR (1985). Genetic evaluation for insect resistance in rice. International Rice Research Institute, Los Baños.

[CR20] Heong KL, Hardy B (eds) (2009) Planthoppers: new threats to the sustainability of intensive rice production systems in Asia. International Rice Research Institute

[CR21] Horgan FG, Arida A, Ardestani G, Almazan MLP (2020). Temperature-dependent oviposition and nymph performance reveal distinct thermal niches of coexisting planthoppers with similar thresholds for development. PLoS One.

[CR22] Howe GA, Jander G (2008). Plant immunity to insect herbivores. Annu Rev Plant Biol.

[CR23] Hu J, Xiao C, He Y (2016). Recent progress on the genetics and molecular breeding of brown planthopper resistance in rice. Rice.

[CR24] International Rice Research Institute (IRRI) (1979). Brown planthopper: Threat to rice production in Asia.

[CR25] International Rice Research Institute (IRRI) (2013). Standard evaluation system for rice.

[CR26] Ishiwatari Y, Honda C, Kawashima I, Nakamura SI, Hirano H, Mori S, Fujiwara T, Hayashi H, Chino M (1995). Thioredoxin h is one of the major proteins in rice phloem sap. Planta.

[CR27] Jain M, Tyagi AK, Khurana JP (2006). Genome-wide analysis, evolutionary expansion, and expression of early auxin-responsive SAUR gene family in rice (*Oryza sativa*). Genomics.

[CR28] Jiang H, Hu J, Li Z, Liu J, Gao G, Zhang Q, Xiao J, He Y (2018). Evaluation and breeding application of six brown planthopper resistance genes in rice maintainer line Jin 23B. Rice.

[CR29] Jones PL, Gacesa P, Butlin RK (1996). Systematics of brown planthopper and related species using nuclear and mitochondrial DNA. Syst Ass.

[CR30] Kang K, Yue L, Xia X, Liu K, Zhang W (2019). Comparative metabolomics analysis of different resistant rice varieties in response to the brown planthopper *Nilaparvata lugens* Hemiptera: Delphacidae. Metabolomics.

[CR31] Khush GS, Brar DS (1991). Genetics of resistance to insects in crop plants. Adv Agron.

[CR32] Kim D, Langmead B, Salzberg SL (2015). HISAT: a fast spliced aligner with low memory requirements. Nat Methods.

[CR33] Kim ST, Cho KS, Kim SG, Kang SY, Kang KY (2003). A rice isoflavone reductase-like gene, *OsIRL*, is induced by rice blast fungal elicitor. Mol Cell.

[CR34] Lee JS, Wissuwa M, Zamora OB, Ismail AM (2018). Novel sources of aus rice for zinc deficiency tolerance identified through association analysis using high-density SNP array. Rice Sci.

[CR35] Li C, Luo C, Zhou Z, Wang R, Ling F, Xiao L, Lin Y, Chen H (2017). Gene expression and plant hormone levels in two contrasting rice genotypes responding to brown planthopper infestation. BMC Plant Biol.

[CR36] Li H, Huang W, Liu ZW, Wang YX, Zhuang J (2016). Transcriptome-based analysis of Dof family transcription factors and their responses to abiotic stress in tea plant (*Camellia sinensis*). Int J Genomics.

[CR37] Li H, Zhou Z, Hua H, Ma W (2020). Comparative transcriptome analysis of defense response of rice to *Nilaparvata lugens* and *Chilo suppressalis* infestation. Int J Biol Macromol.

[CR38] Li N, Wei S, Chen J, Yang F, Kong L, Chen C, Ding X, Chu Z (2018). OsASR 2 regulates the expression of a defence-related gene, Os2H16, by targeting the GT-1 cis-element. Plant Biotechnol J.

[CR39] Ling Y, Weilin Z (2016). Genetic and biochemical mechanisms of rice resistance to planthopper. Plant Cell Rep.

[CR40] Liu Y, Wu H, Chen H, Liu Y, He J, Kang H, Sun Z, Pan G, Wang Q, Hu J, Zhou F (2015). A gene cluster encoding lectin receptor kinases confers broad-spectrum and durable insect resistance in rice. Nat Biotechnol.

[CR41] Livak KJ, Schmittgen TD (2001). Analysis of relative gene expression data using real-time quantitative PCR and the 2(−delta delta C(T)) method. Methods.

[CR42] Martin M (2011). Cutadapt removes adapter sequences from high-throughput sequencing reads. EMBnet J.

[CR43] Naik SB, Divya D, Sahu N, Sundaram RM, Sarao PS, Singh K, Lakshmi VJ, Bentur JS (2018). A new gene *Bph33(t)* conferring resistance to brown planthopper (BPH), *Nilaparvata lugens* (Stål) in rice line RP2068-18-3-5. Euphytica.

[CR44] Nomura K, Melotto M, He SY (2005). Suppression of host defense in compatible plant–*Pseudomonas syringae* interactions. Curr Opin Plant Biol.

[CR45] Okada K, Abe H, Arimura GI (2015). Jasmonates induce both defense responses and communication in monocotyledonous and dicotyledonous plants. Plant Cell Physiol.

[CR46] Otuka A, Matsumura M, Watanabe T, Van Dinh T (2008). A migration analysis for rice planthoppers, *Sogatella furcifera* (Horváth) and *Nilaparvata lugens* (Stål) (Homoptera: Delphacidae), emigrating from northern Vietnam from April to may. Appl Entomol Zool.

[CR47] Pathak MD, Cheng CH, Fortuno ME (1969). Resistance to *Nephotettix impicticeps* and *Nilaparvata lugens* in varieties of rice. Nat.

[CR48] Pu L, Xie G, Ji C, Ling B, Zhang M, Xu D, Zhou G (2012). Transmission characteristics of southern rice black-streaked dwarf virus by rice planthoppers. Crop Prot.

[CR49] Ramesh K, Padmavathi G, Deen R, Pandey MK, Lakshmi VJ, Bentur JS (2014). Whitebacked planthopper *Sogatella furcifera* (Horváth)(Homoptera: Delphacidae) resistance in rice variety Sinna Sivappu. Euphytica.

[CR50] Sama VSAK, Rawat N, Sundaram RM, Himabindu K, Naik BS, Viraktamath BC, Bentur JS (2014). A putative candidate for the recessive gall midge resistance gene *gm3* in rice identified and validated. Theor Appl Genet.

[CR51] Savary S, Willocquet L, Elazegui FA, Castilla NP, Teng PS (2000). Rice pest constraints in tropical Asia: quantification of yield losses due to rice pests in a range of production situations. Plant Dis.

[CR52] Schneider CA, Rasband WS, Eliceiri KW (2012). NIH image to ImageJ: 25 years of image analysis. Nat Methods.

[CR53] Sezer M, Butlin RK (1998). The genetic basis of host plant adaptation in the brown planthopper (*Nilaparvata lugens*). Heredity.

[CR54] Soranzo N, Gorla MS, Mizzi L, De Toma G, Frova C (2004). Organisation and structural evolution of the rice glutathione S-transferase gene family. Mol Gen Genomics.

[CR55] Srinivasan TS, Almazan MLP, Bernal CC, Fujita D, Ramal AF, Yasui H, Subbarayalu MK, Horgan FG (2015). Current utility of the *BPH25* and *BPH26* genes and possibilities for further resistance against plant-and leafhoppers from the donor cultivar ADR52. Appl Entomol Zool.

[CR56] Strickland JA, Orr GL, Walsh TA (1995). Inhibition of Diabrotica larval growth by Patatin, the lipid acyl hydrolase from potato tubers. Plant Physiol.

[CR57] Tan J, Wu Y, Guo J, Li H, Zhu L, Chen R, He G, Du B (2020). A combined microRNA and transcriptome analyses illuminates the resistance response of rice against brown planthopper. BMC Genomics.

[CR58] Thimm O, Bläsing O, Gibon Y, Nagel A, Meyer S, Krüger P, Selbig J, Müller LA, Rhee SY, Stitt M (2004). MAPMAN: a user-driven tool to display genomics data sets onto diagrams of metabolic pathways and other biological processes. Plant J.

[CR59] Thorvaldsdóttir H, Robinson JT, Mesirov JP (2013). Integrative genomics viewer (IGV): high-performance genomics data visualization and exploration. Brief Bioinform.

[CR60] Trapnell C, Roberts A, Goff L, Pertea G, Kim D, Kelley DR, Pimentel H, Salzberg SL, Rinn JL, Pachter L (2012). Differential gene and transcript expression analysis of RNA-seq experiments with TopHat and cufflinks. Nat Protoc.

[CR61] Trapnell C, Williams BA, Pertea G, Mortazavi A, Kwan G, Van Baren MJ, Salzberg SL, Wold BJ, Pachter L (2010). Transcript assembly and quantification by RNA-Seq reveals unannotated transcripts and isoform switching during cell differentiation. Nat Biotechnol.

[CR62] Tukey JW (1953). The problem of multiple comparisons.

[CR63] Usadel B, Nagel A, Thimm O, Redestig H, Blaesing OE, Palacios-Rojas N, Selbig J, Hannemann J, Piques MC, Steinhauser D, Scheible WR (2005). Extension of the visualization tool MapMan to allow statistical analysis of arrays, display of coresponding genes, and comparison with known responses. Plant Physiol.

[CR64] Wang H, Shi S, Guo Q, Nie L, Du B, Chen R, Zhu L, He G (2018). High-resolution mapping of a gene conferring strong antibiosis to brown planthopper and developing resistant near-isogenic lines in 9311 background. Mol Breed.

[CR65] Wang L, Tang N, Gao X, Chang Z, Zhang L, Zhou G, Guo D, Zeng Z, Li W, Akinyemi IA, Yang H (2017). Genome sequence of a rice pest, the white-backed planthopper (*Sogatella furcifera*). Gigascience.

[CR66] Wang XL, He RF, He GC (2005). Construction of suppression subtractive hybridization libraries and identification of brown planthopper-induced genes. J Plant Physiol.

[CR67] Wang Y, Cao L, Zhang Y, Cao C, Liu F, Huang F, Qiu Y, Li R, Luo X (2015). Map-based cloning and characterization of *BPH29*, a B3 domain-containing recessive gene conferring brown planthopper resistance in rice. J Exp Bot.

[CR68] Wang Y, Guo H, Li H, Zhang H, Miao X (2012). Identification of transcription factors potential related to brown planthopper resistance in rice via microarray expression profiling. BMC Genomics.

[CR69] Wei K, Chen H (2018). Global identification, structural analysis and expression characterization of cytochrome P450 monooxygenase superfamily in rice. BMC Genomics.

[CR70] Wei Z, Hu W, Lin Q, Cheng X, Tong M, Zhu L, Chen R, He G (2009). Understanding rice plant resistance to the Brown Planthopper (*Nilaparvata lugens*): a proteomic approach. Proteomics.

[CR71] Wen M, Xie M, He L, Wang Y, Shi S, Tang T (2016). Expression variations of miRNAs and mRNAs in rice (*Oryza sativa*). Genome Biol Evol.

[CR72] Wu J, Baldwin IT (2010). New insights into plant responses to the attack from insect herbivores. Annu Rev Genet.

[CR73] Wu Y, Lv W, Hu L, Rao W, Zeng Y, Zhu L, He Y, He G (2017). Identification and analysis of brown planthopper-responsive microRNAs in resistant and susceptible rice plants. Sci Rep.

[CR74] Xu YX, Xiao MZ, Liu Y, Fu JL, He Y, Jiang DA (2017). The small auxin-up RNA OsSAUR45 affects auxin synthesis and transport in rice. Plant Mol Biol.

[CR75] Xue J, Zhou X, Zhang CX, Yu LL, Fan HW, Wang Z, Xu HJ, Xi Y, Zhu ZR, Zhou WW, Pan PL (2014). Genomes of the rice pest brown planthopper and its endosymbionts reveal complex complementary contributions for host adaptation. Genome Biol.

[CR76] Yang Y, Xu J, Leng Y, Xiong G, Hu J, Zhang G, Huang L, Wang L, Guo L, Li J, Chen F (2014). Quantitative trait loci identification, fine mapping and gene expression profiling for ovicidal response to whitebacked planthopper (*Sogatella furcifera* Horvath) in rice (*Oryza sativa* L.). BMC Plant Biol.

[CR77] Yuan H, Chen X, Zhu L, He G (2005). Identification of genes responsive to brown planthopper *Nilaparvata lugens Stål* (Homoptera: Delphacidae) feeding in rice. Planta.

[CR78] Zhang F, Zhu L, He G (2004). Differential gene expression in response to brown planthopper feeding in rice. J Plant Physiol.

[CR79] Zhao Y, Huang J, Wang Z, Jing S, Wang Y, Ouyang Y, Cai B, Xin XF, Liu X, Zhang C, Pan Y (2016). Allelic diversity in an NLR gene BPH9 enables rice to combat planthopper variation. Proc Natl Acad Sci U S A.

[CR80] Zhou GH, Wen JJ, Cai DJ, Li P, Xu DL, Zhang SG (2008). Southern rice black-streaked dwarf virus: a new proposed Fiji virus species in the family Reoviridae. Chin Sci Bull.

[CR81] Zhou WW, Liang QM, Xu Y, Gurr GM, Bao YY, Zhou XP, Zhang CX, Cheng J, Zhu ZR (2013). Genomic insights into the glutathione S-transferase gene family of two rice planthoppers, *Nilaparvata lugens* (Stål) and *Sogatella furcifera* (Horváth)(Hemiptera: Delphacidae). PLoS One.

