# Supplementary material for: RNA-Sequencing Reveals Differentially Expressed Rice Genes Functionally Associated with Defense against BPH and WBPH in RILs Derived from a Cross between RP2068 and TN1
Source: Rice (N Y). 2021 Mar 6;14:27. doi: 10.1186/s12284-021-00470-3 (PMC7936997; doi:10.1186/s12284-021-00470-3)
Supplement: Supplementary file 3 — Additional file 3: Supplementary Tables S1. Genetic similarities among the selected TR RILs*. Table S2. Overview of raw reads and mapped sequences for all the nine samples. Table S3. Sequence information of primers used for qRT-PCR of the shortlisted DEGs identified from the RNA-seq data [file 12284_2021_470_MOESM3_ESM.docx]

Supplementary Tables

Table S1 Genetic similarities among the selected TR RILS *

|  | TR3RR | TR94RR | TR145RS | TR152SR | TR24SS |
| --- | --- | --- | --- | --- | --- |
| TR3RR | 100 |  |  |  |  |
| TR94RR | 84 | 100 |  |  |  |
| TR145RS | 81 | 79 | 100 |  |  |
| TR152SR | 59 | 59 | 59 | 100 |  |
| TR24SS | 49 | 51 | 44 | 50 | 100 |

^*^As based on 137 polymorphic markers tested; (Sama et al. 2014, Naik et al. 2018, Sahu et al. unpublished) between the parents RP2068 and TN1.

Table S2 Overview of raw reads and mapped sequences for all the nine samples

| Sample_Name | Raw Reads | Processed Reads | % of high quality data | % Aligned reads |
| --- | --- | --- | --- | --- |
| Sample1-Repl-I-BPH | 27198250 | 26515034 | 97.49 | 78.82 |
| Sample2-Repl-II-BPH | 31023949 | 29976741 | 96.62 | 86.65 |
| Sample3-Repl-I-WBPH | 30520533 | 29500940 | 96.66 | 87.51 |
| Sample4-Repl-II-WBPH | 28775593 | 27848461 | 96.78 | 84.6 |
| Sample5-Control-I | 37382969 | 36228120 | 96.91 | 90.71 |
| Sample6-Repl-III-BPH | 26689308 | 26091075 | 97.759 | 55.52% |
| Sample7-Repl-III-WBPH | 41951057 | 40273623 | 96.001 | 42.64% |
| Sample8-Control-II | 22592033 | 22218836 | 98.348 | 40.50% |
| Sample9-Control-III | 26687321 | 26383803 | 95.461 | 41.06% |

Table S3 Sequence information of primers used for qRT-PCR of the shortlisted DEGs identified from the RNA-seq data

| **S.No** | **Locus ID** | **Genes** | **Primer Forward sequence** | **Primer Reverse sequence** |
| --- | --- | --- | --- | --- |
|  | **UPUP** | | | |
| 1 | LOC_Os03g06850 | B3 DNA domain | CGCTGTCAAGAGAACCCTTA | AACTGGACTGCAAAGGTCTG |
| 2 | LOC_Os02g46640 | HSP DNAJ domain | GGCTGCAAAAGTTCAGGTTA | CTGAGGGTTTGAGACGAAGA |
| 3 | LOC_Os04g47190 | Aminotransferase domain | GGATAGCCGTAAGTGCTGAA | AGGGCTCCTCCGTTCTATTA |
| 4 | LOC_Os03g21110 | emp24/gp25L/p24 | GTCCAGCGGTTTGTTTAGAA | ACCACACGGATCAATCACTT |
| 5 | LOC_Os03g08800 | CutA, chloroplast precursor | TAAAAGGGTTTGTTCCCACA | TTGGCCCTCTGTATTCCATA |
| 6 | LOC_Os06g45090 | Expressed protein | TGCAATCTACAGGCTTCTCC | TTGCAACCCTTTGTTGGTAT |
| 7 | LOC_Os05g12170 | Plant-specific domain TIGR01589 | AGCCCGAAAGAACTCAGAAT | ATGCTTGGAGACAGTTCAGC |
| 8 | LOC_Os09g04160 | Expressed protein | CAGGCATGGTTTATGGTAGG | GTCTGACATCTGTGCCATGA |
|  | **DNDN** | | | |
| 9 | LOC_Os07g33440.1 | Cytochrome P450, putative, | GACAGCCAGTCGTCTTCTCC | CGATCCTCGCGAACTCTATC |
| 10 | LOC_Os01g03530 | Multicopper oxidase domain | AGTTCTTCATGGTCGACAGC | ATTTGGGATGGAGGGAATAG |
| 11 | LOC_Os07g03710.1 | SCP-like extracellular protein | GGAAGTACGGCGAGAACATC | GGTCGTACCACTGCTTCTCC |
| 12 | LOC_Os01g50420.1 F | STE_MEKK_ste11_MAP3K.7 - STE kinases | ACAGTTCGCGTAACATAGCC | ACTGCAGATTTCGGTCTTTG |
| 13 | LOC_Os01g73250 | Abscisic acid stress and ripening | CCCACACCGACGACTACTAC | CTGCTGCCTCATCAGGTACT |
| 14 | LOC_Os01g27210 | Glutathione s transferase | ACTATTGGGCAATCGAAGTG | TTCGCATGTTACGCAAATTA |
| 15 | LOC_Os01g73200 | Peroxidase precursor | ACTACGTCGACCTGCAAAAC | CGACGGAGTAGACGTACTGG |
| 16 | LOC_Os09g31430.1 | Os9bglu30 - beta-glucosidase | GGGCTACTTCACGTGGACAT | CCAGTAGCTGGACTCCTTGC |
|  | **UPDN** | | | |
| 17 | LOC_Os03g47140 | Growth regulatory factor | CCAACATTGTCCCTGTTCTC | GCTTTTGTTGTTCTGCTGGT |
| 18 | LOC_Os03g48490 | Centromere protein | GATGATGAGGGACGATATGC | TTCATCCTGGACCTTTTTGA |
| 19 | LOC_Os12g13570.1 | MYB family TF | AAACAAGGAGGCATGGACAC | GCTCAGCCCATTTATTTCCA |
|  | **DNUP** | | | |
| 20 | LOC_Os01g65210 | Proton dependent transport | AGTGGTGAAGGAACACCAAA | GGTACCCAGCTAGGAGAAGC |
| 21 | LOC_Os06g23274 | Zinc finger domain | GGACACTGCATTGACACGTA | CCGCATCTCTCTGATTGATT |
| 22 | LOC_Os10g01044 | Isoflavone reductase | TGAAAACCGATGATCCAACT | AAATGTCACGTGTTCCGTCT |
| 23 | LOC_Os04g52700 | Expressed protein | ACAGCATGCAACACATGAAC | GCCTCTGACGAAATTTGAGA |
| 24 | LOC_Os05g18470 | CRAL/TRIO domain | CAGGTTTGGGTAGAGCAAGA | GACAAGCTGGAGTCTGCATT |
| 25 | LOC_Os01g01650.1 | Isoflavone reductase like, | GTGGATAGCGTGGTGATCCT | CGTATGTGCAGCGTCTTGTT |
| 26 | LOC_Os04g45370.1 | OsSAUR19 Auxin-responsive | CCATCAGAATGGGCTGATTT | CACGCACATGTCGAGAAAAA |
| 27 | LOC_Os11g03440.1 | MYB-like DNA-binding domain | AGATCGGTTCCCATCAACAC | GACTGGAGGTGGACGATGAT |
