# Supplementary figures and images for: RNA-Sequencing Reveals Differentially Expressed Rice Genes Functionally Associated with Defense against BPH and WBPH in RILs Derived from a Cross between RP2068 and TN1
Source: Rice (N Y). 2021 Mar 6;14:27. doi: 10.1186/s12284-021-00470-3 (PMC7936997; doi:10.1186/s12284-021-00470-3)

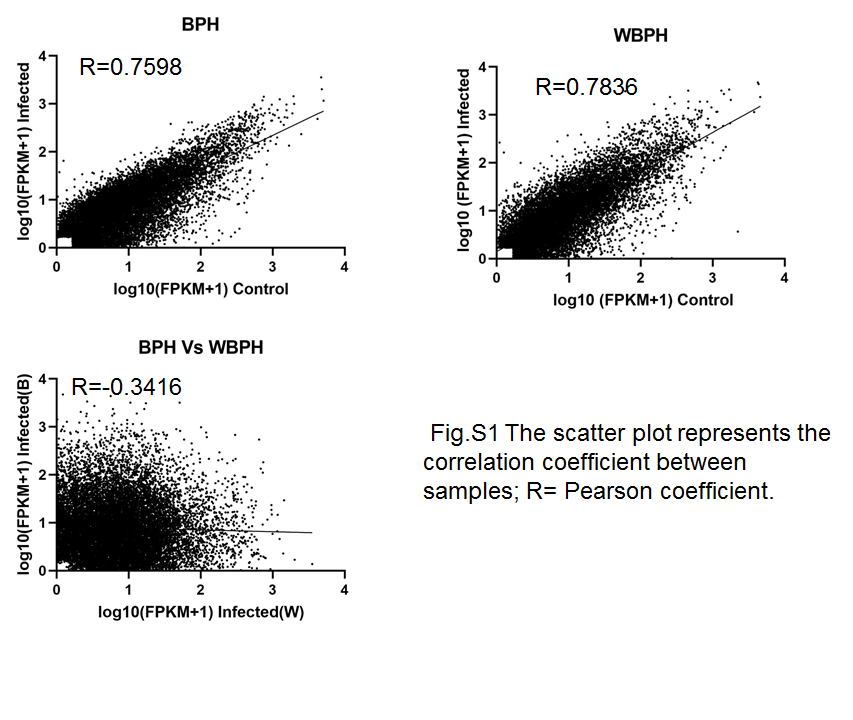

Supplement: Supplementary file 7 — Additional file 7: Supplementary Fig S1. Scatter plot representing correlation coefficient between samples [file 12284_2021_470_MOESM7_ESM.tif]

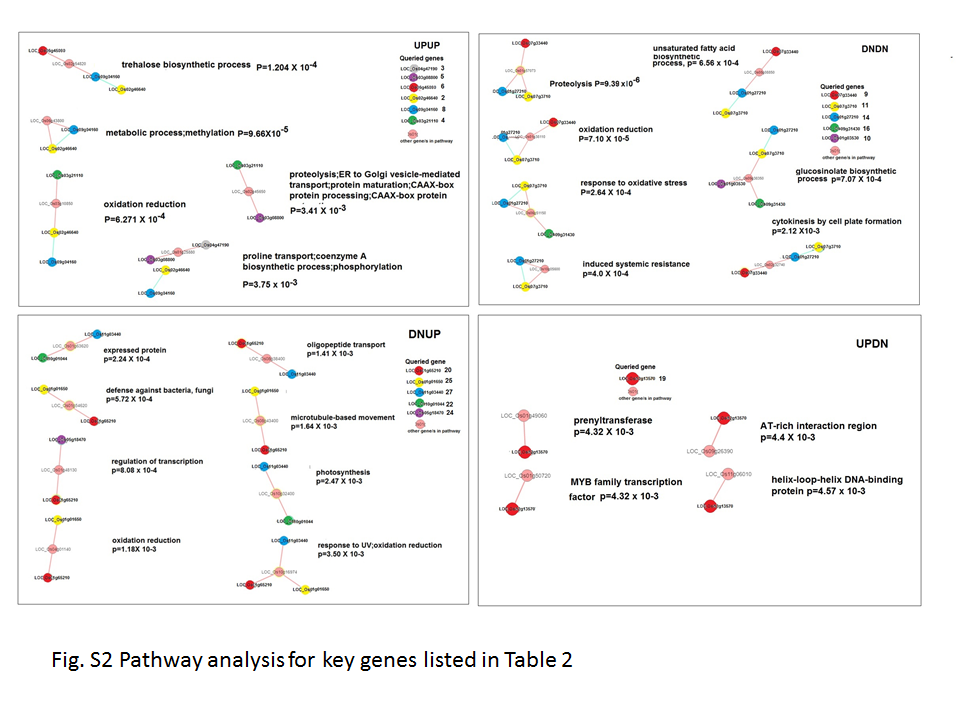

Supplement: Supplementary file 8 — Additional file 8: Supplementary Fig S2. Pathway analysis for key genes listed in Table 2 [file 12284_2021_470_MOESM8_ESM.tif]
